# No Effect of One-Year Treatment with Indomethacin on Alzheimer's Disease Progression: A Randomized Controlled Trial

**DOI:** 10.1371/journal.pone.0001475

**Published:** 2008-01-23

**Authors:** Daniëlle de Jong, René Jansen, Willibrord Hoefnagels, Marja Jellesma-Eggenkamp, Marcel Verbeek, George Borm, Berry Kremer

**Affiliations:** 1 Department of Neurology, Radboud University Nijmegen Medical Center, Nijmegen, The Netherlands; 2 Department of Geriatric Medicine, Radboud University Nijmegen Medical Center, Nijmegen, The Netherlands; 3 Alzheimer Center Nijmegen, Radboud University Nijmegen Medical Center, Nijmegen, The Netherlands; 4 Laboratory of Pediatrics and Neurology, Radboud University Nijmegen Medical Center, Nijmegen, The Netherlands; 5 Department of Epidemiology and Biostatistics, Radboud University Nijmegen Medical Center, Nijmegen, The Netherlands; 6 Department of Geriatric Medicine, Rijnstate Hospital, Arnhem, The Netherlands; University of Washington, United States of America

## Abstract

**Background:**

The objective of this study was to determine whether treatment with the nonselective nonsteroidal anti-inflammatory drug (NSAID) indomethacin slows cognitive decline in patients with Alzheimer's disease (AD).

**Methodology/Principal Findings:**

This double-blind, randomized, placebo-controlled trial was conducted between May 2000 and September 2005 in two hospitals in the Netherlands. 51 patients with mild to moderate AD were enrolled into the study. Patients received 100 mg indomethacin or placebo daily for 12 months. Additionally, all patients received omeprazole. The primary outcome measure was the change from baseline after one year of treatment on the cognitive subscale of the AD Assessment Scale (ADAS-cog). Secondary outcome measures included the Mini-Mental State Examination, the Clinician's Interview Based Impression of Change with caregiver input, the noncognitive subscale of the ADAS, the Neuropsychiatric Inventory, and the Interview for Deterioration in Daily life in Dementia. Considerable recruitment problems of participants were encountered, leading to an underpowered study. In the placebo group, 19 out of 25 patients completed the study, and 19 out of 26 patients in the indomethacin group. The deterioration on the ADAS-cog was less in the indomethacin group (7.8±7.6), than in the placebo group (9.3±10.0). This difference (1.5 points; CI −4.5–7.5) was not statistically significant, and neither were any of the secondary outcome measures.

**Conclusions/Significance:**

The results of this study are inconclusive with respect to the hypothesis that indomethacin slows the progression of AD.

**Trial Registration:**

ClinicalTrials.gov NCT00432081

## Introduction

Early indications that inflammation plays an important role in the pathogenesis of Alzheimer's disease (AD) emerged in 1982, when complement factors were found in senile plaques. [1] Many studies followed that supported the inflammatory hypothesis, and evidence accumulated that anti-inflammatory drugs, in particular nonsteroidal anti-inflammatory drugs (NSAIDs) would either prevent, postpone or treat AD. [2] However, 25 years later, there is still no clinical evidence that NSAIDs have an effect in AD patients, nor is there incontrovertible evidence of the contrary.

In a small randomized controlled trial, the traditional NSAID indomethacin appeared to protect AD patients from cognitive decline. [3] Another small randomized controlled trial studying the effect of diclofenac/misoprostol in AD, found a nonsignificant trend of more advanced deterioration in the placebo group than in the diclofenac/misoprostol group. [4] A large randomized controlled trial with naproxen (440 mg/d) could not confirm the earlier observed trends. [5] Both pilot studies were hampered by high withdrawal rates in the treatment groups due to side effects. Low-dose naproxen was reasonably well tolerated.

The side effects of NSAIDs, e.g. gastrointestinal toxicity, have always been a major concern that limited their use. It was suggested that the beneficial actions of NSAIDs are linked to their ability to inhibit cyclooxygenase-2 (COX-2), while their side effects result from inhibition of COX-1. [6] However, randomized controlled trials with COX-2 selective NSAIDs (rofecoxib, nimesulide, and celecoxib) failed to show an effect on the progression of AD. [5], [7]–[9] Consequently, the traditional nonselective NSAIDs regained interest.

Apart from the promising, but never replicated, results of the initial indomethacin trial, there are also in vitro and animal model studies that support a possible therapeutic effect. Indomethacin inhibited amyloid β (Aβ)-induced neurotoxicity, [10]–[12] and decreased the production of Aβ-protein, interleukin-6, interleukin-1, nitric oxide, and prostaglandin E_2_ in a variety of cultured cells. [13]–[18] Furthermore, indomethacin was found to have anti-amyloidogenic effects in vitro; The formation of Aβ fibrils was dose-dependently inhibited by indomethacin. [19] In rats, indomethacin attenuated microglial infiltration, and improved lipopolysaccharide-induced amnesia. [20], [21] In a transgenic mouse-model of AD-like amyloidosis (Tg2576), indomethacin suppressed brain levels of prostaglandins, [22] and reduced Aβ levels in cortex and hippocampus. [22], [23] This amyloid burden lowering effect was confirmed by other investigators using a combination of indomethacin and vitamin E to treat Tg2576 mice. [24]

Supported by these data, particularly by the prior trial that suggested a therapeutic benefit as well as by its potential Aβ lowering effect, we hypothesized that indomethacin may retard the clinical progression of AD.

## Methods

The protocol for this trial and supporting CONSORT checklist are available as supporting information; see Protocol S1 (Dutch version), Protocol S2 (English version) and Checklist S1.

### Participants

Patients were recruited from May 2000 to August 2004 at the Department of Neurology and at the Memory Clinic, Department of Geriatric Medicine of the Radboud University Nijmegen Medical Center, and at the Memory clinic of the Department of Geriatric Medicine, Rijnstate Hospital, Arnhem, The Netherlands. Patients were eligible if they met the NINCDS/ADRDA criteria for the clinical diagnosis of probable AD, [25] had mild or moderate dementia as measured by a Mini-Mental State Examination (MMSE) [26] score between 10 and 26 inclusive, and were living at home or in a home for the elderly. Patients had to be supported by a reliable caregiver, who accompanied them to each clinic visit in order to provide information about the patient's functional status, and who would ensure that the participants took their test medication.

Patients were excluded if they had a history or current evidence of peptic ulceration; history of gastric surgery or gastrointestinal bleeding; severe and unstable cardiovascular disease; severe pulmonary disease; renal failure (serum creatinine greater than 200 mmol/l); clinically significant liver disease (plasma aspartate and alanine aminotransferase levels three times the upper limit of normal); poorly controlled diabetes mellitus; hypersensitivity to NSAIDs or aspirin; alcohol abuse; or advanced, severe and unstable disease of any type (other than AD), that might interfere with evaluations during the study, including a medical condition which should be expected to progress, recur, or change to such an extent that it might bias the assessment of the clinical or mental status of the patient, or put the patient at special risk. Also, patients taking the following concomitant medications were excluded, because of a possible interaction with indomethacin; aspirin, coumarin derivatives, angiotensin converting enzyme inhibitors, loop diuretics, and long-term use of other NSAIDs or corticosteroids (more than two months immediately before study entry). Intake of the following medication was not allowed during the study because of a possible effect on cognition; estrogen replacement therapy, deprenyl, vitamin E, neuroleptics and anticholinergic medication. Patients using stable doses of cholinesterase inhibitors were eligible, with the provision that the dose should not be changed during the study. Cholinesterase inhibitors could not be initiated during the study.

### Ethics

At both study sites, approval of the local institutional review board to perform the study was received. Informed consent was obtained from each patient and their legally acceptable representative.

### Interventions

The study was a one-year, randomized, double-blinded, placebo-controlled bicenter trial. After screening, patients were randomly assigned to receive 50 mg indomethacin twice daily or placebo twice daily for one year. In addition, patients in both treatment groups received omeprazole 20 mg once daily, to prevent gastrointestinal side effects.

### Objectives

We tested whether indomethacin would have an effect on cognitive and behavioral dysfunction, as well as dysfunction of the activities of daily living, in patients with mild to moderate AD.

### Outcomes

Efficacy was primarily assessed by the cognitive subscale of the AD Assessment Scale (ADAS-cog), [27] an instrument that evaluates memory, language, attention, reasoning, orientation, and praxis (range 0 to 70). Secondary outcome measures included the MMSE, [26] the Clinician's Interview Based Impression of Change with caregiver input (CIBIC+), [28] the noncognitive subscale of the ADAS (ADAS-noncog), [27] the Neuropsychiatric Inventory (NPI), [29], [30] including the NPI caregiver distress scale (NPI-D), [31] and the Interview for Deterioration in Daily life in Dementia (IDDD). [32] The IDDD is a caregiver-based measure, which consists of 20 concretely worded items that reflect the initiative to perform, and the actual performance of self-care and more complex activities.

Cognitive and behavioral assessments were performed at baseline, and at weeks 26 and 52. Safety assessments included vital signs and the recording and rating of any adverse event by the investigator (weeks 4, 8, 12, 26, 38, and 52), physical examination (baseline, week 26, and 52), and routine hematology and chemistry blood tests (baseline, week 4, 8, 26, and 52).

### Sample size

The primary hypothesis tested was that indomethacin would be superior to placebo in retarding cognitive decline as measured on the ADAS-cog after one year of treatment. We aimed at 80% power to detect a 3-point difference in the change in ADAS-cog score after one year between patients who received indomethacin and those who received placebo. ADAS-cog data from previous studies were used in the power calculations for the initial trial, and an SD of 7 was assumed. This yielded a estimated sample size of 67 to be evaluated per group. Since an overall dropout rate of 20% was anticipated, the required sample size was 80 patients per group.

### Randomization – Sequence generation

The statistician provided computer-generated lists of random numbers allocating patients in a 1∶1 ratio to receive indomethacin or placebo. For each center, a separate randomization list was provided.

### Randomization – Allocation concealment

Randomization codes were held by the pharmacy of the Radboud University Nijmegen Medical Center that labeled and dispensed all trial medication. Allocation was concealed from all investigators and patients.

### Randomization – Implementation

Eligible patients were allocated to a randomization number in the same order they were enrolled in the trial at both trial sites. At each visit, patients received a supply of medication (indomethacin or placebo) by the pharmacy, labeled with their randomization number.

### Blinding

The indomethacin and placebo tablets were of identical appearance. Neither the patients nor the investigators knew which treatment they received or dispensed. The blinding process remained complete until all data was entered in the trial database and the accuracy of the data and the database was confirmed. Afterward, the database was forwarded to the statistician for analysis.

### Statistical methods

The changes from baseline in the groups were compared using analysis of covariance with the baseline results of each assessment as a covariate. In an additional analysis, gender and age were added as covariates. Two-sided *p* values and 95% confidence intervals were calculated. The primary efficacy analysis was conducted on the observed values. In addition, the last observation carried forward (LOCF) approach was used.

## Results

### Participant flow and recruitment

**Figure 1:**
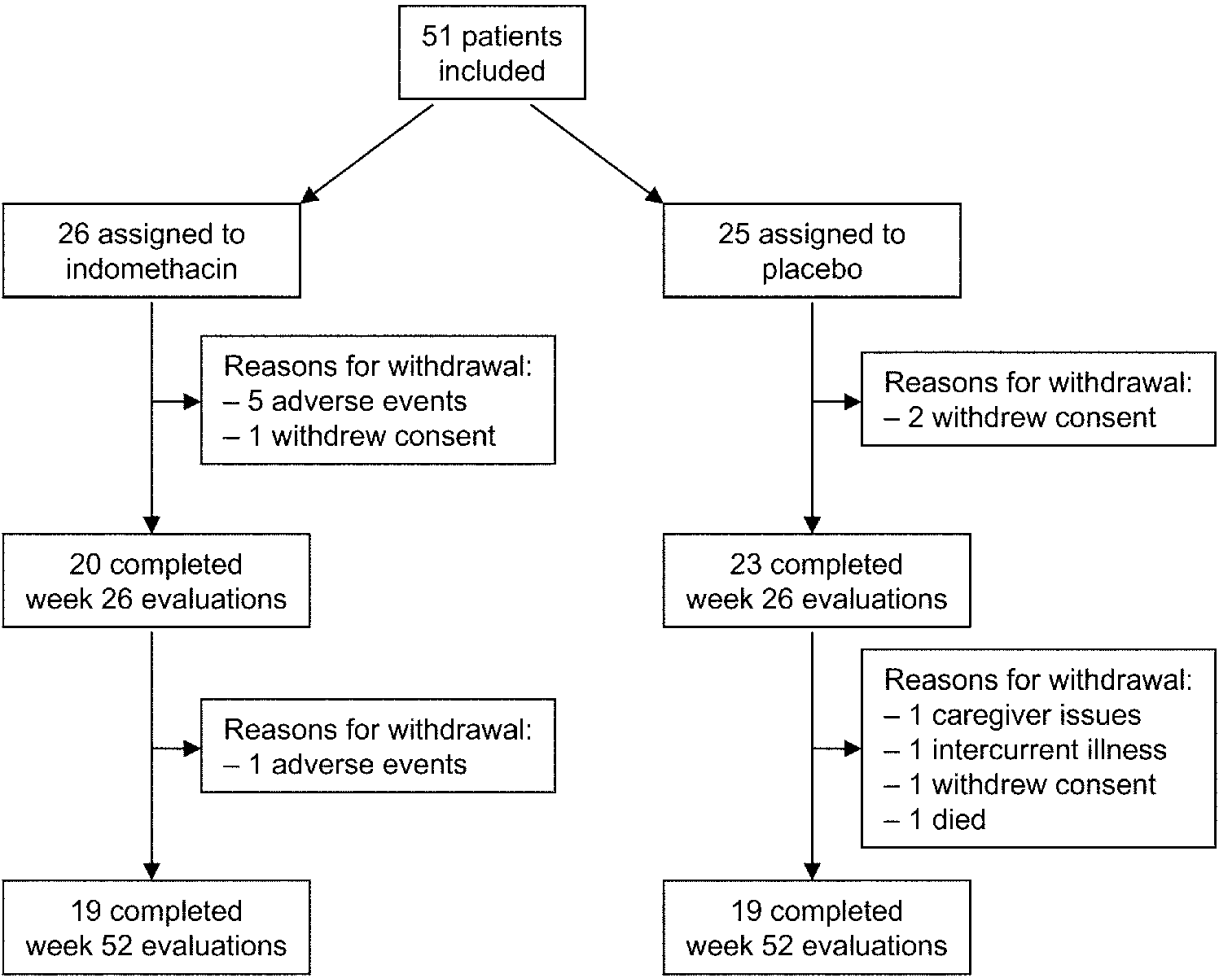
Trial profile.

Figure 1 illustrates the flow of patients through the study protocol. The study was discontinued prematurely after four years, due to difficulties with the enrollment of patients into the study. Based on an inclusion rate of approximately thirteen patients per year, eight more years of enrollment would have been necessary to complete this study. Taking into account scientific, organizational, and financial reasons, the decision was made to discontinue the study. Eventually, fifty-one patients were included in the trial, about one-thirds of the number originally anticipated. Most patients were enrolled at the Memory Clinic, Department of Geriatric Medicine of the Radboud University Nijmegen Medical Center (n = 46), with an inclusion rate of one out of every five to six patients diagnosed with AD. The remainder of patients was enrolled at the outpatient clinic of the Department of Neurology of the Radboud University Nijmegen Medical Center (n = 3), and at the Department of Geriatric Medicine of the Rijnstate Hospital, Arnhem (n = 2).

### Numbers analyzed

Twenty-five patients were randomly assigned to the placebo group, and twenty-six patients to the indomethacin group. Completion rates were 19 of 25 patients (76%) in the placebo group, and 19 of 26 patients (73%) in the indomethacin group. One patient in the indomethacin group discontinued the study in week 48 due to caregiver issues, but completed all week 52 evaluations. The predominant reasons for premature study discontinuation were adverse events (n = 6) in the indomethacin group, and withdrawal of consent (n = 2) in the placebo group. None of the patients that withdrew from the study due to adverse events did complete their follow-up assessments, however all other available assessment data were included in the analysis.

### Baseline data

Treatment groups were similar with respect to demographic and baseline clinical characteristics, except for gender distribution (table 1); in the placebo group 24% of patients were male, and in the indomethacin group 46% of patients. No significant differences were found between baseline assessment scores. Nevertheless, baseline NPI, NPI-D, and ADAS-noncog scores were higher in the indomethacin group, suggesting that patients in this group had more behavioral problems.

**Table 1:** Baseline characteristics of the study population by treatment group.

Characteristics	Placebo (n = 25)	Indomethacin (n = 26)
Men/women	6/19	12/14
Age (SD), years	72.2 (9.0)	72.7 (6.9)
Education level (SD), range 1 to 5*	2.7 (0.9)	2.4 (1.3)
≥1 APOE ε4 allele, n (%)	11 (44%)	13 (50%)
Disease duration (SD), months	31.1 (19.6)	32.9 (17.4)
Use of cholinesterase inhibitor, n (%)	2 (8,0%)	2 (7.7%)
MMSE score (SD)	20.2 (3.9)	19.1 (4.1)
ADAS-cog score (SD)	19.7 (8.8)	20.2 (8.3)
ADAS-noncog score (SD)	2.8 (2.7)	3.5 (3.6)
NPI score (SD)	7.1 (6.7)	11.2 (12.0)
NPI-D score (SD)	5.6 (4.5)	7.7 (7.3)
IDDD score (SD)	21.2 (12.8)	22.8 (13.7)

* level 1 is primary school only; level 5 is university level.

MMSE = Mini-Mental State Examination; ADAS-cog = cognitive subscale of the Alzheimer's Disease Assessment Scale; ADAS-noncog = noncognitive subscale of the ADAS; NPI = Neuropsychiatric Inventory; NPI-D = caregiver distress scale of the NPI; IDDD = Interview for Deterioration in Daily life in Dementia.

### Outcomes, estimation, and ancillary analyses

The effect of treatment on primary and secondary outcome measures is shown in table 2. The decrease in mean ADAS-cog score after one year of therapy was 1.5 points less in the indomethacin group (7.8±7.6) compared to the placebo group (9.3±10.0), however this was not statistically significant (CI −4.5–7.5). When using the LOCF approach to analyze the difference in change in ADAS-cog score, or when gender and age were included as covariate in the analysis, the results were similar to the primary analysis (data not shown).

**Table 2:** Mean change from baseline of outcome measures, and difference in scores between the placebo and indomethacin group, after six months and one year of treatment.

	Placebo group mean change from baseline (SD)		Indomethacin group mean change from baseline (SD)		Difference between groups* (95% CI)	
Measure	6 months (n = 23)	1 year (n = 19)	6 months (n = 20)	1 year (n = 19)	6 months	1 year
ADAS-cog	3.9 (4.5)	9.3 (10.0)	4.8 (5.8)	7.8 (7.6)	−0.9 (−4.1–2.2)	1.5 (−4.5–7.5)
ADAS-noncog	−0.3 (1.5)	1.6 (4.2)	1.5 (4.1)	3.8 (6.7)	−1.8 (−3.9–0.2)	−2.8 (−6.7–1.1)
MMSE	−2.4 (3.6)	−5.4 (5.5)	−2.3 (3.2)	−3.4 (4.3)	0.1 (−1.9–2.1)	1.6 (−1.6–4.8)
NPI	−0.3 (4.9)	9.4 (14.0)	1.7 (14.0)	3.2 (18.1)	−3.6 (−10.1–2.9)	4.6 (−6.6–15.8)
NPI-D	−0.9 (3.5)	6.5 (8.8)	0.7 (6.4)	1.4 (8.3)	−2.2 (−5.4–1.0)	4.6 (−1.3–10.5)
IDDD	10.4 (8.3)	18.2 (14.8)	9.5 (14.4)	19.4 (13.8)	0.8 (−6.4–8.0)	−1.5 (−11.0–8.0)
CIBIC+	5.3 (0.7)	5.7 (0.7)	5.1 (0.8)	5.6 (0.8)	0.2 (−0.2–0.6)	0.1 (−0.3–0.5)

* differences, adjusted for baseline (analysis of covariance).

Negative change in scores from baseline indicates improvement, with the exception of the MMSE score (positive change indicates improvement), and the CIBIC+ (higher score means worse compared to baseline).

Positive difference between groups means in favor of the indomethacin group, for all measures.

ADAS-cog = cognitive subscale of the Alzheimer's Disease Assessment Scale; ADAS-noncog = noncognitive subscale of the ADAS; MMSE = Mini-Mental State Examination; NPI = Neuropsychiatric Inventory; NPI-D = caregiver distress scale of the NPI; IDDD = Interview for Deterioration in Daily life in Dementia; CIBIC+ = Clinician's Interview Based Impression of Change with caregiver input.

The decline of secondary outcome measures after six months or one year of treatment did not show statistically significant differences between groups either (table 2). Additional analysis, using the LOCF approach, showed similar results.

### Adverse Events

Blood test abnormalities, abnormalities found during physical examination, and adverse events reported on case report forms were grouped into categories for analysis. Adverse events that occurred in at least two patients in either treatment group are listed in table 3. Patients in the indomethacin group had more frequent adverse events. Dyspepsia, epigastic pain, or abdominal distress or pain, were reported more frequently in the placebo group (n = 3), than in the indomethacin group (n = 1). In both groups, there were no reports of serious gastrointestinal adverse events, such as gastroenteritis, ulceration or bleeding. Nausea, dizziness, and hyperglycemia were more common in the indomethacin group, whereas diarrhea, constipation, and headache, were more common in the placebo group. Weight loss, defined as 5 percent or more loss of body weight, was seen in three patients in the indomethacin group, and in one patient in the placebo group. New cases of hypertension were reported more frequently in the indomethacin group (5 out of 22 non-hypertensive patients at baseline; 23%), than in the placebo group (2 out of 18 non-hypertensive patients at baseline; 11%). Despite these cases of elevated blood pressure, the change in mean arterial pressure (MAP) during the trial was not significantly different between groups; MAP increased 2.5±10.6 (mean±SD) mmHg in the indomethacin group, and decreased 1.2±9.5 mmHg in the placebo group (p = 0.20).

**Table 3:** Adverse events that occurred in at least two patients in either treatment group.

Adverse event	Placebo and omeprazole (n = 25)	Indomethacin and omeprazole (n = 26)
Nausea	0	2
Diarrhea or constipation	3	2
Dyspepsia, epigastric or abdominal pain	3	1
Weight loss (≥ 5% during the study)	1	3
Headache	2	0
Dizziness	1	3
Hyperglycemia	1	2
Hypertension (new cases)	2	5

Serious adverse events were also more common in the indomethacin group (n = 5) than in the placebo group (n = 1; table 4), and reason for study withdrawal (table 4). In the indomethacin group, blood tests revealed a considerable elevation of creatinine levels (>1.5 times the upper limit of normal) in three patients, without clinical symptoms. All three patients had abnormal creatinine clearance rates before entering the trial, and one of these patients had a history of nephrectomy. After discontinuation of the study, serum creatinine levels returned to their previous levels. Blood tests also revealed increased levels (>3 times the upper limit of normal) of alanine aminotransferase, and aspartate aminotransferase in one patient in the indomethacin group, without clinical symptoms. Liver function tests normalized within four weeks after study discontinuation. Nine days after enrollment in the study, one patient in the indomethacin group had a lacunar stroke. Evaluation after four months of recovery revealed only minor disabilities (increased memory impairment and irritability). Death occurred in one patient in the placebo group after 38 weeks of study participation. The cause of death of this patient is unknown.

**Table 4:** Serious adverse events.

Serious adverse event	Placebo and omeprazole (n = 25)	Indomethacin and omeprazole (n = 26)
Elevated creatinine*	0	3
Abnormal liver function tests†	0	1
Stroke (lacunar)	0	1
Death	1	0

* >1.5 times the upper limit of normal.

† >3 times the upper limit of normal.

## Discussion

### Interpretation

In this study, indomethacin 50 mg twice daily did not show any statistically significant effects on the progression of dementia in patients with mild to moderate AD during a 1-year period, as measured by testing of cognition, behavior, and activities of daily living, and by overall clinical global impression.

Although our study included more patients than the earlier trials with indomethacin and diclofenac/misoprostol, the number of included patients was still too small. [3], [4] Thus, the study was clearly underpowered, resulting in very wide confidence intervals; The confidence interval for the ADAS-cog was 12 points (range –4.5 to 7.5). This means that the difference between the groups should have been at least 6 points to reach statistical significance.

### Generalizability

The enrollment of patients was hampered by the extensive exclusion criteria, especially the exclusion of patients using aspirin, angiotensin converting enzyme inhibitors or loop diuretics. The institutional review board specifically imposed this criterion, since interaction of these drugs with indomethacin might aggravate the occurrence of side effects of indomethacin. Not only did patient enrollment suffer from these strict criteria, it is also responsible for another limitation of the study; Our study population was a highly selected group of AD patients, with no or minor cardiovascular comorbidity, and thus not representative of the average AD population.

### Overall evidence

By its nature our study cannot prove that anti-inflammatory drugs in general and indomethacin in particular are ineffective. However, the study outcome is consistent with earlier trials that investigated prednisone, hydroxychloroquine, and various selective and non-selective NSAIDs in similar designs; All these studies failed to demonstrate a beneficial effect on disease progression. [4], [5], [7]–[9], [33], [34] These failures may have been due to the pharmacokinetic or pharmacological properties of the drugs being used. But it may also be questioned whether anti-inflammatory treatment will ever be efficacious in treating symptomatic AD. Although they may have preventive effects, they may no longer be effective in patients with established disease.

Indomethacin in combination with omeprazole was reasonably well tolerated in this elderly population. There were no serious gastrointestinal tract events. Dyspepsia, epigastic pain, or abdominal distress or pain were more common in the placebo group, and may have been caused by omeprazole, and not by indomethacin. However, elderly patients should be carefully monitored when using indomethacin. Blood pressure should be checked regularly, and blood tests must be done before and during indomethacin treatment. In patients with elevated creatinine clearance, the administration of indomethacin should be avoided.

In conclusion, the results of this study are inconclusive with respect to the hypothesis that indomethacin slows the progression of AD. Owing to its limited statistical power, this study does not alter the conclusions from earlier trials that NSAIDs do not appear to be effective in altering the progression of symptoms in AD. Thus, treatment of AD patients with indomethacin should currently not be recommended, and further treatment trials with NSAIDs in AD patients should be thoroughly reconsidered. However, primary prevention trials with NSAIDs, in particular ibuprofen (in combination with omeprazole), are warranted to further investigate the effect of long-term NSAID use on risk of AD.

## Supporting Information

Checklist S1 CONSORT Checklist
(0.05 MB DOC)

Protocol S1 Trial Protocol (Dutch)
(0.26 MB DOC)

Protocol S2 Trial Protocol (English)
(0.16 MB DOC)
