# Supplementary material for: No Effect of One-Year Treatment with Indomethacin on Alzheimer's Disease Progression: A Randomized Controlled Trial
Source: PLoS One. 2008 Jan 23;3(1):e1475. doi: 10.1371/journal.pone.0001475 (PMC2194921; doi:10.1371/journal.pone.0001475)
Supplement: Protocol S1 — Trial Protocol (Dutch) (0.26 MB DOC) [file pone.0001475.s002.doc]

Protocol

Remming van de progressie van de ziekte van Alzheimer door langdurige toediening van indometacine.

Deelnemers:

Mw. drs. D. de Jong, arts-onderzoeker (1)

Prof.dr. H.P.H. Kremer, neuroloog (1)

Dr. R.W.M.M. Jansen, klinisch geriater (2)

Prof.dr. W. Hoef­nagels, klinisch geriater (2)

M.J.H.Jellesma-Eggenkamp (5)

Dr.ir. M.M. Verbeek, klinisch chemicus (1,3)

Prof.dr. Y. Hekster, ziekenhuisapotheker (4)

Dr. M. van ’t Hof, statisticus (6)

Afdelingen (1) Neurologie, (2) Geria­trie, (3) Laboratorium voor kindergeneeskunde en neurologie, en (4) Klinische Farmacie, Acade­misch Ziekenhuis Nijmegen - St.Rad­boud; (5) Neurologie, Rijnstate Ziekenhuis Arnhem, (5) Klinische farmacie, (6) Afdeling MIES, Faculteit Medi­sche Weten­schap­pen, Katholieke Universi­teit Nijmegen

**INHOUDSOPGAVE**

STUDIE SYNOPSIS

1 Titel onderzoek [4](#__RefHeading___Toc459692875)

2 Introductie [4](#__RefHeading___Toc459692876)

3 Doel en vraagstelling [5](#__RefHeading___Toc459692877)

3.1 Doel van het onderzoek [5](#__RefHeading___Toc459692878)

3.2 Vraagstelling [5](#__RefHeading___Toc459692879)

4 Opzet van het onderzoek [5](#__RefHeading___Toc459692880)

5 Patiëntenselectie [5](#__RefHeading___Toc459692881)

5.1 Aantal patiënten [5](#__RefHeading___Toc459692882)

5.2 Inclusie criteria [5](#__RefHeading___Toc459692883)

5.3 Exclusie criteria [5](#__RefHeading___Toc459692884)

5.4 Stopcriteria [6](#__RefHeading___Toc459692885)

5.5 Diagnostiek voor rekrutering [6](#__RefHeading___Toc459692886)

5.6 Rekrutering van patiënten [6](#__RefHeading___Toc459692887)

5.7 Informatie aan de huisarts [7](#__RefHeading___Toc459692888)

6 Duur van het onderzoek en tijdschema [7](#__RefHeading___Toc459692889)

6.1 Per patiënt (eindpunt) [7](#__RefHeading___Toc459692890)

6.2 Looptijd van het onderzoek [7](#__RefHeading___Toc459692891)

7 Ethische en wettelijke aspecten [7](#__RefHeading___Toc459692892)

7.1 Verklaring van Helsinki [7](#__RefHeading___Toc459692893)

7.2 Good Clinical Practice [7](#__RefHeading___Toc459692894)

7.3 Ethische commissie [7](#__RefHeading___Toc459692895)

7.4 Informed consent [8](#__RefHeading___Toc459692896)

7.5 Privacy [8](#__RefHeading___Toc459692897)

8 Randomisatie [8](#__RefHeading___Toc459692898)

9 Medicatie [8](#__RefHeading___Toc459692899)

9.1 Eigenschappen van indometacine [8](#__RefHeading___Toc459692900)

9.2 Doseerschema [8](#__RefHeading___Toc459692901)

9.3 Productie [8](#__RefHeading___Toc459692902)

9.4 Bijwerkingen, risico en ongemak voor de patiënt [9](#__RefHeading___Toc459692903)

9.5 Additionele medicatie [9](#__RefHeading___Toc459692904)

10 Uitkomst [9](#__RefHeading___Toc459692905)

10.1 Eindpunt [9](#__RefHeading___Toc459692906)

10.2 Primaire uitkomstvariabele [9](#__RefHeading___Toc459692907)

10.3 Secundaire uitkomstvariabelen [9](#__RefHeading___Toc459692908)

10.4 Staken van het onderzoek in het geval van bijwerkingen [10](#__RefHeading___Toc459692909)

11 Bewaking [10](#__RefHeading___Toc459692910)

11.1 Bewaking van bijwerkingen [10](#__RefHeading___Toc459692911)

11.2 Bewaking van de medicatie-inname [10](#__RefHeading___Toc459692912)

12 Statistische bewerking [11](#__RefHeading___Toc459692913)

12.1 Sample grootte en power calculaties [11](#__RefHeading___Toc459692914)

12.2 Stratificatie [11](#__RefHeading___Toc459692915)

12.3 Statistische methode [11](#__RefHeading___Toc459692916)

12.4 Interim analyse [11](#__RefHeading___Toc459692917)

13 Studierecords [11](#__RefHeading___Toc459692918)

13.1 Confidentialiteit [11](#__RefHeading___Toc459692919)

13.2 Bewaren van de studierecords [11](#__RefHeading___Toc459692920)

14 Publicatie van resultaten [12](#__RefHeading___Toc459692921)

15 Organisatie en uitvoering van het onderzoek [12](#__RefHeading___Toc459692922)

15.1 Onderzoekers [12](#__RefHeading___Toc459692923)

15.2 Plaats van uitvoering [12](#__RefHeading___Toc459692924)

16 Referenties [13](#__RefHeading___Toc459692925)

BIJLAGEN I – VI

**STUDIE SYNOPSIS**

| **TITEL** | Remming van de progressie van de ziekte van Alzheimer door langdurige toediening van indometacine. |
| --- | --- |
| **VRAAGSTELLING** | Is er een verschil vast te stellen in het beloop van de cognitieve en gedragsmatige functiestoornissen en stoornissen in dagelijks functioneren bij patiënten met de ziekte van Alzheimer, die langdurig worden behandeld met indometacine, vergeleken met onbehandelde patiënten? |
| **OPZET** | Dubbel-blind, gerandomiseerd, placebo-gecontroleerd multicentrisch onderzoek met parallelle groepen patiënten met de ziekte van Alzheimer (80 patiënten per groep), waarin gedurende een behandeling van 1 jaar het effect van indometacine op de deterioratie van cognitie bestudeerd wordt. |
| **PATIENTEN** | Inclusie criteria: Patiënten met een lichte tot matig ernstige vorm van de ziekte van Alzheimer (DSM-IV en NINCDS/ADRDA criteria, MMSE score: 10 tot en met 26); schriftelijk informed consent aanwezig.  Exclusie criteria: Medische voorgeschiedenis van een ulcus ventriculi of duodeni, maagoperaties, doorgemaakte bloedingen uit de tractus digestivus of bijwerkingen van NSAID’s; het gebruik van salicylaten, coumarinederivaten, NSAID’s, corticosteroïden en acetylcholinesteraseremmers. |
| **DUUR** | De duur van het onderzoek voor de patiënt bedraagt 1 jaar. |
| **EthiEK** | Voor uitvoering van het onderzoek is reeds een verklaring van geen bezwaar afgegeven door de ethische commissie van het AZN St. Radboud. |
| **Medicatie** | Indometacine heeft de volgende gunstige eigenschappen voor de gezochte indicatie: een groot verdelingsvolume, een goede penetratie van bloed-hersenbarriëre en vergelijking met reeds uitgevoerd onderzoek is mogelijk.  Het doseerschema van indometacine is 2 maal daags 50 mg.  Ter voorkoming van mogelijke gastro-intestinale bijwerkingen zal daarnaast een  1 maal daags 20 mg omeprazol worden voorgeschreven. |
| **Uitkomsten** | Primaire uitkomstvariabele: cognitieve deelschaal van de Alzheimer’s Disease Assessment Scale (ADAS-cog). De ADAS schaal wordt veel gebruikt in medicatieonderzoek bij de ziekte van Alzheimer, hetgeen leidt tot onderlinge vergelijkbaarheid.  Secundaire uitkomstvariabelen: De niet-cognitieve deelschaal van de Alzheimer’s Disease Assessment Scale (ADAS-noncog), de Clinician Interview-Based Impression (CIBIC+), de Mini-Mental State Examination (MMSE), de Neuropsychiatric Inventory (NPI) en de IDDD.  Evaluaties vinden plaats tijdens het baseline bezoek, na 26 en na 52 weken. |
| **Bewaking** | Bewaking van de eventuele bijwerkingen geschiedt 4, 8, 12, 26, 38 en 52 weken na start van het onderzoek door middel van een vragenlijst, lichamelijk onderzoek en bloedonderzoek. |
| **Sample grootte en power calculatie** | Deze zijn gebaseerd op de volgende gegevens betreffende de ADAS-cog en op de volgende overwegingen: een deterioratie over een periode van één jaar van 5.0 punten (SD: 7.0); een klinisch relevant verschil tussen de groepen gedefinieerd als een verschil van 3.0 punten of meer; met 80% power en een eenzijdige waarschijnlijkheid van 5% om een van de nulhypothese (geen verschil tussen medicatie- en placebogroep) afwijkende uitkomst te ontdekken.  Gebaseerd op deze overwegingen zijn er per groep 67 patiënten nodig.  Uitgaande van een percentage uitvallers van 20% per groep, bedraagt dan het totale te rekruteren aantal 2 x 80 = 160 patiënten. |

# Titel onderzoek

Remming van de progressie van de ziekte van Alzheimer door langdurige toediening van indometacine.

# Introductie

Er zijn aanwijzingen dat ontstekingsmechanismen een rol spelen bij de neuronale degeneratie bij patiënten met de ziekte van Alzheimer. Deze degeneratie is geassocieerd met deposities van het βA4 eiwit in amyloid (seniele plaques), abnormale fosforylering van intraneuronale tau-eiwitten en aggregatie van neuronale cytoskeleteiwitten (neurofibrillaire tangles), en ophoping van geactiveerde macrofaagachtige cellen, zogenaamde microgliacellen, rond seniele plaques^1;2^.

Los van hetgeen deze processen initieert en hoe deze gebeurtenissen samenhangen, staat momenteel internationaal in de belangstelling de rol van geactiveerde microgliacellen en de door hen geproduceerde cytokines^2-4^. Het acute fase eiwit α1-antichymotrypsine (α1-ACT), diverse complementfactoren (o.a. C1q), het adhesiemolecuul ICAM-1 en cytokines zoals interleukine-6 (IL-6) kunnen in seniele plaques aangetroffen worden^5-8^. In de liquor van Alzheimer patiënten werd een verhoogde activiteit van α1-ACT^9-12^ en verlaagde concentraties interleukine-6 en C1q gevonden^13;14^. Activatie van microgliacellen vindt mogelijk plaats door interactie van βA4 fibrillen met microgliacellen middels een "receptor for advanced glycosylation end-products" (RAGE)^15^.

Een hypothese is dat de cytokines die geproduceerd worden door geactiveerde microglia-cellen, neuronen beschadigen en aldus bijdragen tot de deterioratie van patiënten met de ziekte van Alzheimer. Remming van deze afgifte zou dan deterioratie van de ziekte moeten tegengaan.

Uit retrospectief epidemiologisch onderzoek bleek dat het aantal patiënten met reumatoïde artritis dat tevens leed aan de ziekte van Alzheimer, kleiner was dan te verwachten viel op grond prevalentiegegevens^16^. Dit effect kan mogelijk toegeschreven worden aan het gebruik van anti-inflammatoire middelen. Ook in een studie bij tweelingen werd een verminderd risico op de ziekte van Alzheimer bij gebruik van Non-Steroid Anti Inflammatory Drugs (NSAID's) gevonden^17^. Uit een recent overzicht van 17 studies op dit gebied komt een consistent beeld naar voren van een relatie tussen (vroeger) gebruik van anti-inflammatoire middelen en een verlaagde incidentie van de ziekte van Alzheimer^18^.

Op grond van deze overwegingen werd een dubbelblind onderzoek met een beperkte patiëntengroep opgezet, waarbij gevonden werd dat patiënten met de ziekte van Alzheimer die indometacine toegediend kregen minder snel deterioreerden dan patiënten die een placebo verstrekt kregen^19^.

Het vooruitzicht dat het ontstaan of de progressie van de ziekte van Alzheimer geremd zou kunnen worden met eenvoudig beschikbare en vooral goedkope medicijnen zoals NSAID's, is zeer aantrekkelijk. Echter, het is relatief moeilijk een medicatietrial met deze medicijnen op te zetten, aangezien aan de kant van de geneesmiddelenindustrie geen belangstelling bestaat voor dergelijke medicijnen waarvan het patent vrijwel altijd al verstreken is.

# Doel en vraagstelling

## Doel van het onderzoek

Vaststellen of de progressie van de ziekte van Alzheimer vertraagd kan worden door het chronische gebruik van NSAID's.

## Vraagstelling

Is er een verschil vast te stellen in het beloop van de cognitieve en gedragsmatige functiestoornissen en stoornissen in dagelijks functioneren bij patiënten met de ziekte van Alzheimer, die langdurig worden behandeld met indometacine, vergeleken met onbehandelde patiënten?

# Opzet van het onderzoek

Dubbel-blind, gerandomiseerd, placebo-gecontroleerd multicentrisch onderzoek met parallelle groepen patiënten met de ziekte van Alzheimer (80 patiënten per groep), waarin gedurende een behandeling van 1 jaar het effect van indometacine op de deterioratie van cognitie bestudeerd wordt.

# Patiëntenselectie

## Aantal patiënten

Voor het onderzoek zullen 160 patiënten gerekruteerd moeten worden: 80 voor de medicatiegroep en 80 voor de placebogroep (zie 12.2: Sample grootte en power calculaties).

Voor de jaarlijks te rekruteren aantallen patiënten: zie 6.2.

## Inclusie criteria

- Patiënt voldoet aan de DSM-IV criteria voor dementie van het Alzheimer type (zie bijlage I)^20^.
- Patiënt voldoet aan de NINCDS/ADRDA criteria voor "probable Alzheimer’s Disease” (zie bijlage I)^21^.
- MMSE score van 10 t/m 26^22^.
- Patiënt is thuis of in het verzorgingshuis woonachtig.
- Familielid of verzorger beschikbaar voor voldoende informatie over het functioneren van de patiënt.
- Schriftelijk informed consent aanwezig (patiënt of wettelijk vertegenwoordiger).

## Exclusie criteria

- De aanwezigheid van NINCDS/ADRDA criteria die de diagnose "probable Alzheimer’s Disease” onzeker dan wel onwaarschijnlijk maken (zie bijlage I).
- De aanwezigheid van NINDS-AIREN criteria die de diagnose vasculaire dementie waarschijnlijk maken (zie bijlage I)^23^.
- Ernstige bijwerking of overgevoeligheidsreactie op een NSAID in de anamnese.
- Ulcus ventriculi of duodeni (aangetoond), maagoperatie of doorgemaakte bloeding uit de tractus digestivus in de medische voorgeschiedenis.
- Actief ulcus ventriculi of duodeni.
- Tekenen van manifest hartfalen (decompensatio cordis).
- Ernstige pulmonale aandoening (O_2_-suppletie, i.v. steroïden, theophylline of FEV_1_ < 60% van de verwachte waarde).
- Ontregelde Diabetes Mellitus (HbA_1c_ ≥ 10% totale Hb of glucose > 15 mmol/l).
- Nierfunctiestoornissen (kreatinine > 200 μmol/l).
- Leverfunctiestoornissen (ASAT, ALAT, γGt > 3 x maximaal normale waarde).
- Het gebruik van salicylaten of coumarinederivaten.
- Het gebruik van ACE remmers en/of lisdiuretica.
- Het dagelijkse gebruik van de volgende geneesmiddelen langer dan 2 maanden direct voorafgaand aan het onderzoek of tijdens het onderzoek (vanwege een additief ulcerogeen effect en mogelijke effecten op de primaire respons variabele):
- NSAID's.
- Corticosteroïden (systemische toedieningsvorm).
- Het gebruik van medicatie waarvan beschreven is dat er mogelijk een effect bestaat op de cognitie:
- oestrogenen (orale toedieningsvorm).
- vitamine E (≥ 50 mg per dag).
- langwerkende benzodiazepinen (bijv. flurazepam, nitrazepam) of kort-werkende benzodiazepinen > 10 mg per dag (bijv. oxazepam, temazepam).
- antipsychotica.
- lithium.
- antidepressiva.
- anticholinergica.
  (Het gebruik van acetylcholinesteraseremmers tijdens het onderzoek is toegestaan, mits er sprake is van een stabiele dosering)
- Het overmatig gebruik van alcohol (meer dan 3 eenheden per dag).
- Patiënten die niet in staat zijn de medicatie betrouwbaar in te nemen, of waar geen verzorgende bij betrokken is die medicatie-inname kan controleren.

## Stopcriteria

- Patiënt weigert verdere participatie in het onderzoek.
- Optreden van bekende, ernstige bijwerkingen die aan indometacine kunnen worden toegeschreven (maagdarmstoornissen, nierfunctiestoornissen, stoornissen van het bloedbeeld en gestoorde leverfunctiewaarden), of zodanige bijwerkingen van de voorgeschreven medicatie dat door de arts en/of patiënt het voortgezet gebruik van het middel ongewenst wordt geacht.
- Patiënten bij wie therapietrouw niet meer te garanderen is.
- Noodzaak voor systemisch corticosteroïd-gebruik langer dan twee weken tijdens de looptijd van het onderzoek.
- Intercurrente ziekten of aandoeningen zoals beschreven in de exclusiecriteria.
- Een zodanig beloop van de aandoening, dat de diagnose ziekte van Alzheimer niet langer waarschijnlijk wordt geacht.
- Het verbreken van de medicatiecode om welke reden dan ook.

## Diagnostiek voor rekrutering

Voor de initiële diagnose "waarschijnlijke ziekte van Alzheimer" zal gebruik gemaakt worden van de zgn. NINDS/ADRDA criteria (zie bijlage I)^21^. Middels een checklist zal gekeken worden welk screeningsonderzoek inmiddels bij de patiënt verricht is en wat de uitkomst van dat onderzoek is. Ontbrekende onderdelen van het screenings-onderzoek zullen worden aangevuld alvorens tot rekrutering over te gaan (zie ook bijlage II en III).

## Rekrutering van patiënten

Deelnemers zullen worden gerekruteerd uit de volgende groepen:

- Patiënten die verwezen worden naar de polikliniek Geriatrie of Neurologie en die voldoen aan de inclusiecriteria.
- Patiënten uit het facultaire wetenschappelijke onderzoek "Diagnostiek dementie in de 1e en 2e lijn" (Geriatrie/WOK/HSV).

Gelet op het aantal per jaar te rekruteren patiënten kan het aanbod uit deze twee bronnen echter onvoldoende zijn. Daarom zal, indien nodig, participatie worden gevraagd van:

- Regionale ziekenhuizen: CWZ te Nijmegen en Rijnstate Ziekenhuis te Arnhem.
- Regionale verpleegtehuizen.
- Huisartsen.

Indien ook dit onvoldoende rekrutering oplevert, zal overgegaan worden tot werving via een oproep in de regionale nieuwsmedia.

## Informatie aan de huisarts

Bij inclusie in de trial wordt de huisarts op de hoogte gesteld van deelname van de patiënt aan het wetenschappelijke onderzoek via een informatiebrief (zie bijlage IV).

# Duur van het onderzoek en tijdschema

## Per patiënt (eindpunt)

De duur van het onderzoek voor de patiënt, van initiële rekrutering tot uiteindelijk de laatste beoordeling, bedraagt 1 jaar. Gedurende dat jaar zullen verschillende beoordelingen plaats vinden (zie evaluatieschema in bijlage III).

## Looptijd van het onderzoek

De looptijd van het totale onderzoek, van de rekrutering van de eerste patiënten tot de uiteindelijke analyse van de resultaten, zal 4 jaar bedragen.

Voor de rekrutering van patiënten zal het volgende tijdschema worden aangehouden:

- Jaar 1: 55 patiënten te rekruteren.
- Jaar 2: 55 patiënten te rekruteren.
- Jaar 3: 50 patiënten te rekruteren.
- Jaar 4: vervolgen van de laatste 50 patiënten en analyse, bewerking en publicatie van de data aan het einde van jaar 4 of het begin van jaar 5.

# Ethische en wettelijke aspecten

## Verklaring van Helsinki

Het onderzoek zal worden uitgevoerd in volledige overeenstemming met de principes van de "Verklaring van Helsinki" (amendementen in Tokyo, Venetië, Hong Kong).

## Good Clinical Practice

De onderzoekers zullen opzet, uitvoering en bewaking van het onderzoek verrichten in overeenstemming met de eisen voor Good Clinical Practice.

## Ethische commissie

Het onderzoek zal ter beoordeling worden voorgelegd aan de ethische commissie; pas indien deze een verklaring van geen bezwaar heeft afgegeven, zal met de rekrutering en inclusie van patiënten begonnen worden.

## Informed consent

Naast een mondelinge uiteenzetting zal de patiënt en zijn/haar verzorgende ook schriftelijk geïnformeerd worden over doel, aard, uitvoering en risico's van het onderzoek (zie bijlage V), en gevraagd zal worden aan de patiënt om een instemmingverklaring te tekenen (zie bijlage VIa). In het geval van wilsonbekwame patiënten zal plaatsvervangend toestemming worden gevraagd aan de wettelijk vertegenwoordiger van de patiënt, of indien deze ontbreekt, de schriftelijk gemachtigde, of indien deze ontbreekt, de echtgenoot of andere levensgezel (zie bijlage VIb). Deze zal volledig worden geïnformeerd over alle relevante aspecten van het klinische onderzoek.

## Privacy

De eisen die gelden vanuit de Wet Persoonsregistratie worden gehanteerd om zorgvuldig om te kunnen gaan met de persoonsgegevens van de patiënten die zijn opgenomen in deze studie. Voor de instemming van de patiënt wordt verwezen naar de instemmingverklaring. Rapportage aan personen buiten de direct betrokken groep onderzoekers zal plaatsvinden op basis van geanonimiseerde gegevens.

# Randomisatie

Nieuwe deelnemende patiënten aan het onderzoek zullen een sequentieel trialnummer toegewezen krijgen. De lijst met mogelijke trialnummers is vooraf gekoppeld aan een gerandomiseerde lijst met medicatienummers, overeenkomend met 80 batches indometacine en 80 batches placebo. De deelnemende apotheker zal de codesleutel beheren. Deze procedure garandeert een exact evenwichtige initiële randomisatie. Bij differentiële uitval later in de trial zal echter een verschil in aantal deelnemers tussen beide groepen ontstaan. In de uiteindelijke analyse zal het aantal uitvallers in de indometacine-groep ("intention to treat") een belangrijk gegeven zijn.

# Medicatie

## Eigenschappen van indometacine

Indometacine is een in Nederland geregistreerd geneesmiddel. Het middel is niet geregistreerd voor de te onderzoeken indicatie. Hoewel indometacine niet meer gezien wordt als eerste keus NSAID bij reumatische aandoeningen, kent het middel de volgende gunstige eigenschappen voor de gezochte indicatie:

- groot verdelingsvolume
- goede penetratie van bloed-hersenbarriëre
- vergelijking met reeds uitgevoerd onderzoek is mogelijk

## Doseerschema

Het doseerschema van indometacine is als volgt: 2 maal daags 50 mg.

Naast indometacine wordt een maal daags 20 mg omeprazol voorgeschreven.

## Productie

Voor dit onderzoek, waarvoor naar berekening in totaal 4480 capsules indometacine 25 mg/ placebo, 112.320 capsules indometacine 50 mg/ placebo en 58.400 capsules omeprazol nodig zijn, zullen bij een producent van generieke geneesmiddelen de batches indometacine (capsules 25 mg) en placebo, omeprazol (capsules 20 mg) besteld worden.

## Bijwerkingen, risico en ongemak voor de patiënt

Het gebruik van NSAID's leidt bij meer dan 20% van de ouderen tot gastro-intestinale bijwerkingen. Het risico van ernstige gastro-intestinale complicaties kan meer dan 10 maal verhoogd zijn (odds ratio), vooral in de eerste 3 maanden van behandeling, bij mensen boven de 60 jaar, bij een middel als indometacine, bij hogere doseringen en bij het gelijktijdig gebruik van corticosteroïden^24^.

Ter voorkoming van mogelijke gastro-intestinale bijwerkingen zal standaard omeprazol worden gegeven^25-27^.

De belasting voor de patiënten wordt laag ingeschat als er geen hinderlijke bijwerkingen van de medicatie optreden. Een verdere (minimale) belasting voor de patiënt vormen regelmatige venapuncties.

## Additionele medicatie

Bestaande medicatie wordt gehandhaafd.

Bepaalde medicijnen vormen reden tot exclusie (zie 5.3).

# Uitkomst

## Eindpunt

Cognitieve status 12 maanden (één jaar) na start van de interventie.

## Primaire uitkomstvariabele

Cognitieve deelschaal van de Alzheimer’s Disease Assessment Scale (ADAS-cog)^28^: Gekozen werd voor de cognitieve deelschaal van de Alzheimer's Disease Assessment Scale. Het gaat hier om 9 cognitieve items van de originele ADAS (maximum score: 48), aangevuld met twee geheugen items (maximum score 22), leidend tot een totale score variërend van 0 tot 70. De geheugen items (word recall en word recognition) worden vooral gebruikt in trials met cholinesteraseremmers, daar deze twee tests vooral gevoelig voor cholinerge manipulatie zouden zijn^29^. De ADAS schaal wordt veel gebruikt in medicatie-onderzoek bij de ziekte van Alzheimer, hetgeen leidt tot onderlinge vergelijkbaarheid. Het is speciaal ontworpen om ziekteprogressie te meten, heeft bij de door ons geselecteerde groep Alzheimer patiënten geen floor/ceilingeffecten, is goed gekarakteriseerd voor wat betreft interrater en test-retest betrouwbaarheid en is relatief snel af te nemen (45 minuten). Power calculaties voor wat betreft deze trial zijn gebaseerd op deze schaal.

## Secundaire uitkomstvariabelen

Niet-cognitieve deelschaal van de Alzheimer’s Disease Assessment Scale (ADAS-noncog)^28^: Het gedeelte van de Alzheimer Disease Assessment Scale dat de niet-cognitieve items uit de oorspronkelijke ADAS bevat. Dit meet psychiatrische (vooral stemmingsgerelateerde) aspecten en motore veranderingen in patiënten.

Clinician Interview-Based Impression (CIBI)^30^: Een zeer globale schaal om verandering over de tijd gemeten vanaf een baseline te meten. De 7-punts schaal varieert van 1 (zeer veel verbeterd) via 4 (geen verandering) tot 7 (zeer veel verslechterd). Deze schaal laat aan de beoordelende clinicus een grote mate van vrijheid aan welke klinische aspecten hij of zij aandacht wil besteden bij de initiële beoordeling, met uitsluiting van formele psychometrische tests, maar vereist een consequente en consistente herbeoordeling bij volgende gelegenheden. Inbreng van familieleden en verzorgenden wordt expliciet aangemoedigd. Een dergelijke schaal wordt door de Amerikaanse Food and Drug Administration vereist in medicatie trials voor de ziekte van Alzheimer.

Mini-Mental State Examination (MMSE)^22^: Een veel gebruikte, snelle meting over een groot traject van cognitieve aspecten. Met name geschikt voor het meten van ziekteprogressie.

Neuropsychiatric Inventory (NPI) en NPI-D: Dit meet psychiatrische (vooral stemmingsgerelateerde) veranderingen en gedragsproblemen in patiënten.

Interview for Deterioration in Daily living activities in Dementia (IDDD): Meet beperkingen in het dagelijks leven (ADL en IADL functies).

Bijwerkingen Checklist: ter opsporing van mogelijke bijwerkingen.

## Staken van het onderzoek in het geval van bijwerkingen

Wanneer een ernstige bijwerking optreedt die mogelijk aan indometacine kan worden toegeschreven, zal de trialmedicatie door de dan behandelend arts (huisarts of specialist) op eigen initiatief gestaakt moeten worden. De trial stopt dan voor die patiënt, maar daar de code niet verbroken wordt, zullen de gegevens van de patiënt soms wel in de uiteindelijke analyse van de primaire uitkomst opgenomen kunnen worden. Aan de behandelaren zal gevraagd worden deze beslissing om te stoppen zo snel mogelijk aan de uitvoerenden van het onderzoek door te gegeven.

Bij dubieuze bijwerkingen mag de behandelend arts de medicatie verlagen, zo nodig voor de verdere duur van het onderzoek. Ook hier wordt de code niet verbroken; de patiënt wordt beschouwd als normaal participerend in het onderzoek.

Bij ernstige bijwerkingen kan de behandelend arts aan de toeziend apotheker om opheldering vragen over de gebruikte medicatie. De code is dan verbroken, en de patiënt kan niet meer meedoen aan de uiteindelijke uitkomstanalyse. Dit betekent dat de medicatie ook gestaakt zal worden. Wel zullen, volgens het principe van "intention to treat", de gegevens van de patiënt bij de uiteindelijke analyse betrokken worden.

# Bewaking

## Bewaking van bijwerkingen

De bewaking van de bijwerkingen geschiedt door middel van regelmatige medische controles door een ander dan de geblindeerde onderzoeker van de patiënt:

- Lichamelijk onderzoek: bloeddruk, lichaamsgewicht, tekenen van vochtretentie en oedeemvorming.
- Bloedonderzoek: hemoglobine, leukocyten, thrombocyten, ureum, kreatinine, electrolyten, glucose en leverfuncties.

Een interim analyse op bijwerkingen zal twee keer per jaar plaatsvinden (zie 12.4).

## Bewaking van de medicatie-inname

Afgifte van medicatie met boekhouding van verstrekte aantallen zal de onderzoekers in staat stellen zich een oordeel te vormen over de frequentie van inname. De onderzoeker zal bij ieder bezoek van de patiënt, of bij ieder bezoek aan de patiënt, de aantallen overgebleven medicijnen natellen. Tevens zullen aan de verzorgende(n) van de patiënt vragen gesteld worden over medicatietrouw.

# Statistische bewerking

## Sample grootte en power calculaties

Deze zijn gebaseerd op de volgende gegevens betreffende de ADAS-cog 22 en op de volgende overwegingen: een deterioratie over een periode van één jaar van 5.0 punten (SD: 7.0); een klinisch relevant verschil tussen de groepen gedefinieerd als een verschil van 3.0 punten of meer; met 80% power en een eenzijdige waarschijnlijkheid van 5% om een van de nulhypothese (geen verschil tussen medicatie- en placebogroep) afwijkende uitkomst te ontdekken.

Gebaseerd op deze overwegingen zijn er per groep 67 patiënten nodig. Uitgaande van een percentage uitvallers van 20% per groep, bedraagt dan het totale te rekruteren aantal 2 x 80 = 160 patiënten.

## Stratificatie

Bij initiële randomisatie zal geen verdere stratificatie plaats vinden. In de uiteindelijke analyse zullen de placebogroep en de medicatiegroep echter vergeleken worden voor wat betreft: leeftijd; geslacht; duur van de ziekte; ernst van de ziekte (gedefinieerd als ADAS-cog/11 score); en ApoE4 fenotype.

## Statistische methode

In de uiteindelijke analyse zal een zogenaamde "evaluatie op increment" plaatsvinden. Van iedere patiënt zal dan de verandering in ADAS-cog/11 score (de primaire uitkomstvariabele) bekeken worden, met vergelijking van de resultaten tussen de twee groepen, middels een 2 x 2 ANOVA (categorieën: groep, tijd; α=0.05).

Secundaire variabelen zullen op vergelijkbare wijze geanalyseerd worden, doch met correctie voor multiple vergelijkingen.

## Interim analyse

Een onafhankelijke commissie zal 2 maal per jaar gedecodeerde gegevens over mogelijke bijwerkingen van de trialmedicatie analyseren, om eventuele onaanvaardbare problemen met deze medicatie in een vroeg stadium op het spoor te komen. Een interim analyse naar het beoogde effect van de trialmedicatie zal, gelet op de grootte van de te onderzoeken groep en de grootte van de te verwachten effecten, niet verricht worden.

# Studierecords

## Confidentialiteit

De studierecords zullen beschouwd worden als medische gegevens. Ze zullen dan ook vallen onder de regels van confidentialiteit, als geldend voor medische gegevens in het algemeen. De records zullen tot tien jaar na het staken van het onderzoek bewaard worden.

## Bewaren van de studierecords

Gedurende de looptijd van het onderzoek zullen de studierecords centraal bewaard worden door de arts-onderzoeker. De gegevens bevatten: baseline gegevens omtrent de patiënt; uitkomsten van scorelijsten; registratie van bijwerkingen.

# Publicatie van resultaten

De resultaten van dit onderzoek zullen voor publicatie worden aangeboden aan peer-reviewed vakbladen. Anonimiteit en privacy van de betrokken patiënten zullen hierbij volledig gewaarborgd worden. Indien het onderzoek resultaten oplevert die een brede maatschappelijke relevantie hebben, zal voor publicaties in de lekenpers contact worden opgenomen met de afdeling “PR en Voorlichting”, ten einde publiciteit op verantwoorde wijze te verzorgen**.**

Tenminste alle in dit voorstel genoemde medewerkenden aan het onderzoek zullen als co-auteurs bij de publicaties voortkomend uit dit onderzoek vermeld worden.

# Organisatie en uitvoering van het onderzoek

## Onderzoekers

Coördinatie:

Mw. drs. D. de Jong, arts-onderzoeker.

Onderzoekers, betrokken bij de opstelling, verdere ontwikkeling, en realisatie van het protocol:

Prof.dr. W. Hoefnagels, klinisch geriater.

Dr. R.W.M.M. Jansen, klinisch geriater.

Prof.dr. H.P.H. Kremer, neuroloog.

Mw. drs. D. de Jong, arts-onderzoeker.

Dr.ir. M. Verbeek, chemicus.

Prof.dr. Y. Hekster, ziekenhuisapotheker.

Dr. M.A. van 't Hof, statisticus.

Uitvoerenden, betrokken bij de beoordeling van patiënten:

Mw. drs. D. de Jong, arts-onderzoeker.

Dr. R.W.M.M. Jansen.

Prof. dr. W.H.L. Hoefnagels.

Andere arts werkzaam op de geheugenpolikliniek van de afdeling Geriatrie.

Rekrutering van patiënten:

Mw. D. de Jong.

Monitoring van bijwerkingen:

Behandelend arts.

Prof.dr. Y. Hekster.

Codering medicatie/farmacie:

Prof.dr. Y. Hekster.

Statistische bewerking:

Dr. M.A. van ’t Hof.

## Plaats van uitvoering

Patiënten zullen aan het begin van het onderzoek voor de allereerste beoordeling gezien worden op de polikliniek geriatrie ("geheugenpoli") van het Radboud Ziekenhuis. Latere beoordelingen zullen plaats vinden of op deze polikliniek, of thuis bij de patiënt, dan wel in het verpleegtehuis. Een en ander afhankelijk van de toestand van de patiënt.

# Referenties

1. Selkoe DJ. Alzheimer's disease: a central role for amyloid. J.Neuropathol.Exp.Neurol. 1994;53:438-447.

2. Eikelenboom P, Zhan SS, van Gool WA, Allsop D. Inflammatory mechanisms in Alzheimer's disease. Trends.Pharmacol.Sci. 1994;15:447-450.

3. Klegeris A, Walker DG, McGeer PL. Activation of macrophages by Alzheimer beta amyloid peptide. Biochem.Biophys.Res.Commun. 1994;199:984-991.

4. McGeer PL, Rogers J, McGeer EG. Neuroimmune mechanisms in Alzheimer disease pathogenesis. Alzheimer.Dis.Assoc.Disord. 1994;8:149-158.

5. Verbeek MM, Otte Holler I, Westphal JR, Wesseling P, Ruiter DJ, de Waal RM. Accumulation of intercellular adhesion molecule-1 in senile plaques in brain tissue of patients with Alzheimer's disease. Am.J.Pathol. 1994;144:104-116.

6. Abraham CR, Selkoe DJ, Potter H. Immunochemical identification of the serine protease inhibitor alpha 1-antichymotrypsin in the brain amyloid deposits of Alzheimer's disease. Cell 1988;52:487-501.

7. Eikelenboom P, Stam FC. Immunoglobulins and complement factors in senile plaques. An immunoperoxidase study. Acta Neuropathol.Berl. 1982;57:239-242.

8. Strauss S, Bauer J, Ganter U, Jonas U, Berger M, Volk B. Detection of interleukin-6 and alpha 2-macroglobulin immunoreactivity in cortex and hippocampus of Alzheimer's disease patients. Lab.Invest. 1992;66:223-230.

9. Harigaya Y, Shoji M, Nakamura T, Matsubara E, Hosoda K, Hirai S. Alpha 1-antichymotrypsin level in cerebrospinal fluid is closely associated with late onset Alzheimer's disease. Intern.Med. 1995;34:481-484.

10. Licastro F, Parnetti L, Morini MC, et al. Acute phase reactant alpha 1-antichymotrypsin is increased in cerebrospinal fluid and serum of patients with probable Alzheimer disease. Alzheimer.Dis.Assoc.Disord. 1995;9:112-118.

11. Matsubara E, Hirai S, Amari M, et al. Alpha 1-antichymotrypsin as a possible biochemical marker for Alzheimer-type dementia. Ann.Neurol. 1990;28:561-567.

12. Brugge K, Katzman R, Hill LR, Hansen LA, Saitoh T. Serological alpha 1-antichymotrypsin in Down's syndrome and Alzheimer's disease. Ann.Neurol. 1992;32:193-197.

13. Yamada K, Kono K, Umegaki H, et al. Decreased interleukin-6 level in the cerebrospinal fluid of patients with Alzheimer-type dementia. Neurosci.Lett. 1995;186:219-221.

14. Smyth MD, Cribbs DH, Tenner AJ, et al. Decreased levels of C1q in cerebrospinal fluid of living Alzheimer patients correlate with disease state. Neurobiol.Aging 1994;15:609-614.

15. Yan SD, Chen X, Fu J, et al. RAGE and amyloid-beta peptide neurotoxicity in Alzheimer’s disease. Nature 1996;382:685-691.

16. Andersen K, Launer LJ, Ott A, Hoes AW, Breteler MM, Hofman A. Do nonsteroidal anti-inflammatory drugs decrease the risk for Alzheimer's disease? The Rotterdam Study. Neurology 1995;45:1441-1445.

17. Breitner JC, Gau BA, Welsh KA, et al. Inverse association of anti-inflammatory treatments and Alzheimer's disease: initial results of a co-twin control study. Neurology 1994;44:227-232.

18. McGeer PL, Schulzer M, McGeer EG. Arthritis and anti-inflammatory agents as possible protective factors for Alzheimer's disease: a review of 17 epidemiologic studies. Neurology 1996;47:425-432.

19. Rogers J, Kirby LC, Hempelman SR, et al. Clinical trial of indomethacin in Alzheimer's disease. Neurology 1993;43:1609-1611.

20. American Psychiatric Association. Diagnostic and statistical manual of mental disorders. 4th ed. Washington, DC:American Psychiatric Press, 1994:

21. McKhann G, Drachman D, Folstein M, Katzman R, Price D, Stadlan EM. Clinical diagnosis of Alzheimer's disease: report of the NINCDS-ADRDA Work Group under the auspices of Department of Health and Human Services Task Force on Alzheimer's Disease. Neurology 1984;34:939-944.

22. Folstein MF, Folstein SE, McHugh PR. "Mini-mental state". A practical method for grading the cognitive state of patients for the clinician. J Psychiatr Res 1975;12:189-198.

23. Roman GC, Tatemichi TK, Erkinjuntti T, et al. Vascular dementia: diagnostic criteria for research studies. Report of the NINDS-AIREN International Workshop. Neurology 1993;43:250-260.

24. Gabriel SE, Jaakkimainen L, Bombardier C. Risk for serious gastrointestinal complications related to use of nonsteroidal anti-inflammatory drugs. A meta-analysis. Ann.Intern.Med. 1991;115:787-796.

25. Hawkey CJ, Karrasch JA, Szczepanski L, et al. Omeprazole compared with misoprostol for ulcers associated with nonsteroidal antiinflammatory drugs. Omeprazole versus Misoprostol for NSAID-induced Ulcer Management (OMNIUM) Study Group. N.Engl.J.Med. 1998;338:727-734.

26. Yeomans ND, Tulassay Z, Juhasz L, et al. A comparison of omeprazole with ranitidine for ulcers associated with nonsteroidal antiinflammatory drugs. Acid Suppression Trial: Ranitidine versus Omeprazole for NSAID-associated Ulcer Treatment (ASTRONAUT) Study Group. N.Engl.J.Med. 1998;338:719-726.

27. Langtry HD, Wilde MI. Omeprazole. A review of its use in Helicobacter pylori infection, gastro-oesophageal reflux disease and peptic ulcers induced by nonsteroidal anti-inflammatory drugs. Drugs 1998;56:447-486.

28. Rosen WG, Mohs RC, Davis KL. A new rating scale for Alzheimer's disease. Am.J.Psychiatry 1984;141:1356-1364.

29. Davis KL, Thal LJ, Gamzu ER, et al. A double-blind, placebo-controlled multicenter study of tacrine for Alzheimer's disease. The Tacrine Collaborative Study Group. N.Engl.J.Med. 1992;327:1253-1259.

30. Knopman DS, Knapp MJ, Gracon SI, Davis CS. The Clinician Interview-Based Impression (CIBI): a clinician's global change rating scale in Alzheimer's disease. Neurology 1994;44:2315-2321.

**BIJLAGE I DIAGNOSTISCHE CRITERIA EN SCREENINGSONDERZOEK**

**DSM-IV criteria voor dementie van het Alzheimer type:**

1. De ontwikkeling van de multipele cognitieve functiestoornissen gekenmerkt door:
2. Geheugenstoornis (verminderd vermogen om nieuwe informatie te leren of zich eerder geleerde informatie te herinneren).
3. Eén (of meer) van de volgende cognitieve stoornissen: afasie, apraxie, agnosie, stoornis van de uitvoerende functies.
4. De cognitieve functiestoornissen van criterium A1 en A2 veroorzaken elk een belangrijke beperking in het sociaal of beroepsmatig functioneren en betekenen een belangrijke achteruitgang ten opzichte van het vroegere functioneren.
5. Het beloop wordt gekarakteriseerd door een geleidelijk ontstaan en een voortdurende cognitieve achteruitgang.
6. De cognitieve functiestoornissen van criterium A1 en A2 worden niet veroorzaakt door een van de volgende aandoeningen:
7. Andere aandoeningen van het centrale zenuwstelsel die progressieve stoornissen in geheugen en cognitie veroorzaken (cerebrovasculaire aandoeningen, ziekte van Parkinson of Huntington, subduraal hematoom, hersentumor, normal-pressure hydrocephalus, e.d.).
8. Systemische aandoeningen waarvan bekend is dat ze dementie kunnen veroorzaken (hypothyreoidie, vitamine B_12_ *< 150 μmol/l*, foliumzuur deficiëntie, neurosyphilis, hypercalciaemie, HIV-infectie, e.d.).
9. Alcohol/drugs misbruik.
10. De stoornissen treden niet uitsluitend op tijdens een delier.
11. De stoornis kan niet beter worden geclassificeerd door een andere As-1 stoornis (depressieve stoornis, schizofrenie, e.d.).

**NINCDS/ADRDA criteria voor "probable Alzheimer’s disease":**

1. Dementie, klinisch vastgesteld, gedocumenteerd door de MMSE of een dergelijke test en bevestigd door neuropsychologisch onderzoek *(bijv. CAMCOG, Trail Making Test, 15 woordentest, Geriatrische Depressie Schaal).*
2. Defecten in twee of meer gebieden van cognitie.
3. Progressieve achteruitgang van geheugen en andere cognitieve functies.
4. Afwezigheid van bewustzijnsstoornis.
5. Beginleeftijd tussen 40 en 90 jaar, meestal na het 65ste jaar.
6. Afwezigheid van systemische of andere neurologische aandoening die een mogelijke oorzaak zouden kunnen zijn van progressieve stoornissen van geheugen en cognitie.

*De diagnose "probable Alzheimer’s disease" wordt ondersteund door:*

- progressieve achteruitgang van specifieke cognitieve functies zoals aphasie, apraxie en agnosie.
- stoornissen in ADL-functies en gedragsveranderingen.
- positieve familie-anamnese.
- aanvullend onderzoek: normale standaardbepalingen van de liquor, normaal of non-specifiek EEG, aanwijzingen voor cerebrale atrofie op CT of MRI met door middel van herhaalde beeldvorming gedocumenteerde progressie.

*De volgende kenmerken maken de diagnose van "probable Alzheimer’s disease" onzeker of onwaarschijnlijk en zal patiënten uit de studie excluderen:*

- een plotseling, apoplectisch begin.
- focale neurologische afwijkingen, zoals hemiparese, sensibiliteitsstoornissen, gezichtsvelduitval en coördinatiestoornissen, vroeg in het begin van de ziekte.
- epilepsie of loopstoornissen in het begin of vroeg in het beloop van de ziekte.

**NINDS-AIREN criteria voor “probable” vasculaire dementie:**

1. *Criteria voor de klinische diagnose “probable” vasculaire dementie bestaan uit alle van de volgende:*
2. Aanwezigheid van dementie, gedefinieerd als:
   cognitieve achteruitgang, gekenmerkt door stoornissen van het geheugen en twee of meer andere cognitieve functies, die interfereren met het dagelijks functioneren en bij voorkeur klinisch zijn vastgesteld en gedocumenteerd met behulp van neuropsychologisch onderzoek.
3. Aanwezigheid van cerebrovasculaire ziekte, gedefinieerd als:

- aanwezigheid van focale afwijkingen bij neurologisch onderzoek, zoals hemiparese, centrale facialis parese, pathologische voetzoolreflex, sensibele stoornissen, hemianopsie en dysarthrie, die verklaard zouden kunnen worden door een CVA (met of zonder voorgeschiedenis hiervan).
- aanwezigheid van relevante cerebrovasculaire afwijkingen bij beeldvormend onderzoek *(CT scan of MRI),* zoals multipele infarcten van de grote vaten *(> 2)* of één enkel strategisch geplaatst infarct (gyrus angularis, thalamus, basaal in de frontaal kwab of in het gebied van de arteria cerebri posterior of anterior), multipele lacunaire infacten *(> 2)* in basale ganglia en witte stof of uitgebreide periventriculaire witte stof laesies (een kwart of meer van de witte stof) of een combinatie hiervan.

1. Aanwezigheid van een relatie tussen A en B, bevestigd door één van de volgende kenmerken:
2. begin van de dementie binnen drie maanden na een klinisch vastgesteld CVA.
3. abrupt begin van de achteruitgang van de cognitieve functies of stapsgewijze toename van de cognitieve stoornissen.

*II. Klinische symptomen overeenkomend met de diagnose “probable vascular dementia”:*

1. loopstoornissen vroeg in het beloop van de ziekte .
2. voorgeschiedenis met balansstoornissen en frequent spontaan vallen.
3. urologische klachten (frequente mictie, urge e.d.) vroeg in het beloop van de ziekte, zonder onderliggende urologische aandoening.
4. pseudobulbaire stoornissen.
5. persoonlijkheids- en stemmingsveranderingen, willoosheid, depressie, emotionele incontinentie en andere subcorticale stoornissen, zoals psychomotore retardatie en stoornissen van de uitvoerende functies.

bijlage II Screeningsonderzoek

*Gedurende 1 maand voorafgaand aan het baseline bezoek:*

1. Anamnese, algemeen intern onderzoek (inclusief lengte en gewicht) en neurologisch onderzoek.
2. Aanvullend onderzoek:
3. Laboratoriumonderzoek:

- Hb, Ht, trombocyten, leukocyten en differentiatie, natrium, kalium, ureum, kreatinine, alkalische fosfatase, ASAT, ALAT, γGT, LDH en glucose.
- Apolipoproteïne E; indien nog niet eerder verricht.
- BSE, calcium, albumine, TSH, vitaminen B1, B6, B12 en foliumzuur; indien > 6 maanden geleden verricht.
- HIV en lues; op indicatie.

1. Urine sediment en kweek.
2. Visusonderzoek en audiometrie; op indicatie.
3. Cerebrale CT scan of MRI; indien nog niet eerder of meer dan 2 jaar geleden verricht, of indicatie voor herhaling.
4. Mini-Mental State Examination (MMSE)

**BIJLAGE III**

**Evaluatie Schema**

|  | **Screening** | **Baseline** |  |  |  |  |  |  |
| --- | --- | --- | --- | --- | --- | --- | --- | --- |
| **Evaluaties** | **Week -1 t/m -4** | **Week 0** | **Week 4** | **Week 8** | **Week 12** | **Week 26** | **Week 38** | **Week 52** |
| NINDS/ADRDA criteria | X |  |  |  |  |  |  |  |
| DSM-IV criteria | X |  |  |  |  |  |  |  |
| NINDS/AIREN criteria | X | (X)^1^ |  |  |  |  |  |  |
| Voorgeschiedenis | X |  |  |  |  |  |  |  |
| Huidige medicatie | X |  |  |  |  |  |  |  |
| Algemeen intern onderzoek | X |  | X |  |  | X |  | X |
| RR/pols/gewicht | X | X | X | X | X | X | X | X |
| Neurologisch onderzoek | X |  |  |  |  | X |  | X |
| Laboratorium onderzoek | X ^2^ |  | X | X |  | X |  | X |
| Urine onderzoek | X |  |  |  |  |  |  |  |
| Visusonderzoek/audiometrie | (X)^3^ |  |  |  |  |  |  |  |
| CT/MRI-cerebrum | (X)^4^ |  |  |  |  |  |  |  |
| MMSE | X |  |  |  |  | X |  | X |
| In/exclusie criteria (overige) | X | X ^5^ |  |  |  |  |  |  |
| Informed consent |  | X |  |  |  |  |  |  |
| Randomisatienummer |  | X |  |  |  |  |  |  |
| ADAS-cog |  | X |  |  |  | X |  | X |
| ADAS-nogcog |  | X |  |  |  | X |  | X |
| CIBIC |  | X |  |  |  | X |  | X |
| FIM |  | X |  |  |  | X |  | X |
| APO-E fenotypering | (X)^6^ |  |  |  |  |  |  |  |
| Checklist bijwerkingen |  |  | X | X | X | X | X | X |
| Indometacine uitgifte + rapportage |  | X | X | X | X | X | X |  |
| Indometacine dagboek |  | X | X | X | X | X | X | X |
| Veranderingen medicatie |  | X | X | X | X | X | X | X |
| Veranderingen medische conditie |  | X | X | X | X | X | X | X |

1. evaluatie NINDS/AIREN criteria eventueel tijdens baselinebezoek, afhankelijk van de beoordeling van de CT/MRI.
2. voor programma laboratoriumonderzoek tijdens screeningsbezoek; zie bijlage II.
3. visusonderzoek/audiometrie alleen op indicatie.
4. CT/MRI-cerebrum alleen indien nog niet eerder of meer dan 2 jaar geleden verricht, of indien er een indicatie is voor herhaling.
5. afronding van in- en exclusiecriteria na bekendwording van alle uitslagen van onderzoek.
6. APO-E fenotypering, indien nog niet eerder verricht.

**BIJLAGE IV INFORMATIE AAN DE HUISARTS**

Betreft: naam patiënt

adres

geboortedatum

Geachte collega,

Bovengenoemde patiënt neemt deel aan een dubbelblind, placebogecontroleerd onderzoek naar het effect van indometacine op het geheugen en algemeen dagelijks functioneren bij patiënten met de ziekte van Alzheimer. Gedurende twaalf maanden zal uw patiënt twee maal daags 50 mg indometacine dan wel placebo gebruiken. Daarnaast wordt een maal daags 20 mg omeprazol voorgeschreven om gastro-intestinale bijwerkingen te voorkomen. In verband met het onderzoek en eventuele bijwerkingen bij het gebruik van indometacine zal uw patiënt regelmatig op de polikliniek Geriatrie worden gecontroleerd.

Op grond van eerder verricht epidemiologisch onderzoek verwachten wij dat anti-inflammatoire middelen de progressie van de ziekte van Alzheimer remmen. Desgewenst zijn wij graag bereid meer informatie te verstrekken omtrent de achtergronden.

Mochten er zich onverwacht problemen voordoen, waarbij het noodzakelijk is te weten of uw patiënt indometacine dan wel placebo gebruikt, dan kunt u te allen tijde contact opnemen met ondergetekenden of de dienstdoende klinisch geriater via onderstaand telefoonnummer. De randomisatiecode kan dan eventueel worden verbroken.

Uw patiënt heeft randomisatienummer: …

Met collegiale hoogachting,

mede namens prof.dr. H.P.H. Kremer, neuroloog,

Mw. D. de Jong, Dr. R.W.M.M. Jansen,

arts-onderzoeker klinisch geriater

Seinnummer *2265 Seinnummer *1050

Telefoonnummer (buiten kantooruren): 024-3616776

**BIJLAGE V INFORMATIE VOOR DE PATIENT**

**Onderzoek naar het effect van het geneesmiddel indometacine op het beloop van de ziekte van Alzheimer.**

Inleiding

U bent gevraagd om deel te nemen aan een wetenschappelijk onderzoek naar het effect van een geneesmiddel met de naam indometacine. Indometacine is geen nieuw middel, want het wordt al heel lang door vele patiënten met reuma gebruikt. Uit diverse onderzoeken bij reumapatiënten is echter gebleken, dat dit geneesmiddel mogelijk het proces van de ziekte van Alzheimer vertraagt. Zodoende is bij een klein aantal Alzheimer patiënten het effect van dit geneesmiddel onderzocht, waarbij dit vermoeden werd bevestigd.

Doel van het onderzoek

Het doel van dit wetenschappelijk onderzoek is te bestuderen of het gebruik van indometacine de voortgang van de ziekte van Alzheimer kan remmen, bij patiënten met lichte tot matig ernstige verschijnselen van die aandoening. Om met behulp van deze onderzoeksopzet betrouwbare uitspraken te kunnen doen, zijn in totaal ongeveer 160 patiënten nodig, die gedurende één jaar het medicijn gebruiken.

Verloop van het onderzoek

Voorafgaand aan dit onderzoek wordt u uitvoerig onderzocht op de polikliniek Geriatrie. Er wordt lichamelijk onderzoek, bloed- en urinetesten gedaan, er volgt een CT-scan van de hersenen (indien deze nog niet eerder is verricht) en een psychologisch onderzoek van de geheugenfuncties. Als alle uitslagen goed zijn, maar er wel sprake is van de ziekte van Alzheimer, kunt u aan het onderzoek deelnemen.

Vervolgens zullen tijdens het gebruik van het geneesmiddel nog ongeveer zes bezoeken (gedurende één jaar) aan de polikliniek volgen, om enkele testen te doen die te maken hebben met onder andere geheugen, oriëntatie en concentratie. Er wordt dan ook gevraagd naar lichamelijke klachten, er wordt lichamelijk onderzoek gedaan en het bloed wordt gecontroleerd op mogelijke bijwerkingen van het geneesmiddel.

Het geneesmiddel

Gedurende één jaar zult u capsules, met of zonder indometacine, gaan gebruiken. De helft van de patiënten krijgt dus capsules die geen werkzame stof bevatten (placebo capsules). Dit is nodig om na afloop van de studie betrouwbare uitspraken te kunnen doen over het werkelijke effect van het medicijn.

Er bestaat een kans dat u bijwerkingen krijgt van indometacine. De bijwerkingen die het meest optreden zijn misselijkheid, diarree, verminderde eetlust, hoofdpijn en duizeligheid. Omdat tevens bekend is dat het maagbloedingen kan geven, krijgt u er een medicijn bij dat de maag zoveel mogelijk beschermt en waarmee we deze bijwerking hopen te voorkomen. Mocht u toch symptomen van een maagbloeding hebben, bijvoorbeeld zwarte ontlasting, dient u onmiddellijk contact op te nemen met de onderzoekers of de dienstdoende klinisch geriater via onderstaand telefoonnummer.

Voorwaarden voor deelname

Om vast te stellen of u aan het onderzoek kunt deelnemen zijn er een aantal voorwaarden opgesteld die vooral te maken hebben met uw algehele conditie, eventuele bijkomende ziekten, gebruik van bepaalde medicijnen, uw woonsituatie en de aanwezigheid van een partner of vaste begeleider.

Ongemakken

De risico's die aan dit onderzoek verbonden zijn hangen samen met de mogelijke bijwerkingen van het medicijn en eventuele complicaties bij afnemen van bloed.

Vergoeding

De reiskosten die gemaakt worden voor deelname aan deze studie worden vergoed.

Uw rechten

Het onderzoek is beoordeeld door de Commissie voor Wetenschappelijk Onderzoek met Mensen van het St. Radboud Ziekenhuis te Nijmegen. Indien u deelneemt aan het onderzoek wordt u gevraagd een verklaring te ondertekenen waarin uw rechten en plichten zijn vastgelegd. Bij eventuele schade als gevolg van het onderzoek kunt u het ziekenhuis aansprakelijk stellen. Het ziekenhuis is hiervoor verzekerd.

Berichtgeving aan de huisarts

Vanaf het moment dat u deelneemt aan dit wetenschappelijke onderzoek wordt uw huisarts daarvan op de hoogte gesteld.

Vragen

Mocht u voor of tijdens deelname aan het onderzoek nog vragen hebben, dan kunt u contact opnemen met de onderzoekers (Mw. D. de Jong, arts-onderzoeker en dr. R.W.M.M. Jansen, klinisch geriater) of met de dienstdoende klinisch geriater (buiten kantooruren) via de polikliniek Geriatrie, telefoonnummer 024-3636776.

**BIJLAGE VIa INFORMED CONSENT PATIENT**

**Onderzoek naar het effect van het geneesmiddel indometacine op het beloop van de ziekte van Alzheimer.**

Mijn behandelend arts, ……………….. heeft mij uitgelegd wat de voor- en nadelen, risico's en ongemakken van bovenvermeld onderzoek zijn. Ik heb de schriftelijke informatie gelezen en de gelegenheid gehad om vragen te stellen. Ik heb redelijk de tijd gehad om een en ander te overdenken. Ik begrijp wat de aard en het doel van dit onderzoek is.

Ik begrijp, dat deelname aan het onderzoek vrijwillig is en dat ik mij op elk moment zonder opgave van redenen uit dit onderzoek kan terugtrekken. Als ik dit doe, zal dit geen enkele invloed hebben op de voor mijn ziekte gebruikelijke behandeling en op de zorg van mijn behandelend arts.

Ik weet, dat voor dit onderzoek relevante medische gegevens over mij gebruikt worden voor wetenschappelijk onderzoek en eventueel gepubliceerd worden. Hiermee stem ik in, mits mijn privacy gewaarborgd wordt.

Mijn behandelend arts, ...............…...mag ter controle van de verzamelde gegevens inzage in relevante delen van mijn medische dossier verstrekken aan andere daartoe bevoegde autoriteiten, op voorwaarde dat hij/zij er voor in staat, dat de vertrouwelijkheid van deze gegevens niet zal en kan worden geschonden door deze personen.

| Ik stem toe deel te nemen aan bovengenoemd onderzoek: | | | | | | | | | | | |
| --- | --- | --- | --- | --- | --- | --- | --- | --- | --- | --- | --- |
|  | | | | | | | | | | | |
| Achternaam en voorletters: | | | | |  | | |  | | | |
| Geboortedatum: | |  |  |  | |  | | | | | |
|  |  |  | |  | | | | | |
|  | | | | | | | | | | | |
| Handtekening: |  | | | | | | Datum: | |  |  |  |
|  |  |  |
|  | | | | | | | | | | | |

| Ondergetekende verklaart dat de hierboven genoemde patiënt over het bovenvermelde onderzoek geïnformeerd is: | | | | | | | |
| --- | --- | --- | --- | --- | --- | --- | --- |
|  | | | | | | | |
| Naam: |  | | |  | | | |
| Functie: |  | | |  | | | |
|  | | | | | | | |
| Handtekening: | |  | Datum: | |  |  |  |
|  |  |  |
|  | | | | | | | |

**BIJLAGE VIb INFORMED CONSENT vertegenwoordiger**

**Onderzoek naar het effect van het geneesmiddel indometacine op het beloop van de ziekte van Alzheimer.**

De behandelend arts, ……………….. heeft mij uitgelegd wat de voor- en nadelen, risico's en ongemakken van bovenvermeld onderzoek zijn. Ik heb de schriftelijke informatie gelezen en de gelegenheid gehad om vragen te stellen. Ik heb redelijk de tijd gehad om een en ander te overdenken. Ik begrijp wat de aard en het doel van dit onderzoek is.

Ik begrijp, dat deelname aan het onderzoek vrijwillig is en dat men zich op elk moment zonder opgave van redenen uit dit onderzoek kan terugtrekken. Als men dit doet, zal dit geen enkele invloed hebben op de voor de ziekte gebruikelijke behandeling en op de zorg van de behandelend arts.

Ik weet, dat voor dit onderzoek relevante medische gegevens gebruikt worden voor wetenschappelijk onderzoek en eventueel gepubliceerd worden. Hiermee stem ik in, mits de privacy gewaarborgd wordt.

De behandelend arts, ...............…...mag ter controle van de verzamelde gegevens inzage in relevante delen van het medische dossier verstrekken aan andere daartoe bevoegde autoriteiten, op voorwaarde dat hij/zij er voor in staat, dat de vertrouwelijkheid van deze gegevens niet zal en kan worden geschonden door deze personen.

| Mij is gevraagd om toestemming te verlenen voor deelname van: | | | | | | |
| --- | --- | --- | --- | --- | --- | --- |
|  | | | | | | |
| Achternaam en voorletters: | | | |  | |  |
| Geboortedatum: |  |  |  | |  | |
|  |  |  | |  | |
|  | | | | | | |
| aan bovenvermeld onderzoek. | | | | | | |
|  | | | | | | |

| Ik stem toe met deelname van bovengenoemde persoon aan dit onderzoek: | | | | | | | |
| --- | --- | --- | --- | --- | --- | --- | --- |
|  | | | | | | | |
| Achternaam en voorletters: | |  | |  | | | |
| Relatie tot de deelnemer: | |  | |  | | | |
|  | | | | | | | |
| Handtekening: |  | | Datum: | |  |  |  |
|  |  |  |
|  | | | | | | | |

| Ondergetekende verklaart dat de hierboven genoemde personen over het bovenvermelde onderzoek geïnformeerd zijn: | | | | | | | |
| --- | --- | --- | --- | --- | --- | --- | --- |
|  | | | | | | | |
| Naam: |  | | |  | | | |
| Functie: |  | | |  | | | |
|  | | | | | | | |
| Handtekening: | |  | Datum: | |  |  |  |
|  |  |  |
|  | | | | | | | |
