# Supplementary material for: No Effect of One-Year Treatment with Indomethacin on Alzheimer's Disease Progression: A Randomized Controlled Trial
Source: PLoS One. 2008 Jan 23;3(1):e1475. doi: 10.1371/journal.pone.0001475 (PMC2194921; doi:10.1371/journal.pone.0001475)
Supplement: Protocol S2 — Trial Protocol (English) (0.16 MB DOC) [file pone.0001475.s003.doc]

Clinical Study Protocol

Effect of indomethacin on the progression of Alzheimer’s disease.

A randomized double blind, placebo-controlled, multicenter clinical trial

Principal Investigators:

H.P.H. Kremer, M.D., Ph.D. (1),

R.W.M.M. Jansen, M.D., Ph.D. (2)

Co-investigators:

D. de Jong, M.D., research fellow (1)

W.H.L. Hoefnagels, M.D., Ph.D. (2), M.J.H.Jellesma-Eggenkamp, M.D. (6)

M.M. Verbeek, Ph.D. (3)

Y. Hekster, Ph.D. (4), M. van ’t Hof, M.Sc., Ph.D. (5)

Departments (1) Neurology, (2) Geria­tric Medicine, (3) Pathology, and (4) Clinical Pharmacology, Radboud University Medical Center Nijmegen; (5) Medical Statistics, Universi­ty of Nijmegen, (6) Rijnstate Hospital, Arnhem, The Netherlands

**TABLE OF CONTENTS**

1 Study title [3](#__RefHeading___Toc444936048)

2 Introduction [3](#__RefHeading___Toc444936049)

3 Study objectives [3](#__RefHeading___Toc444936050)

3.1 Primary objective [3](#__RefHeading___Toc444936051)

3.2 Secondary objectives [4](#__RefHeading___Toc444936052)

4 Study design [4](#__RefHeading___Toc444936053)

5 Patient selection [4](#__RefHeading___Toc444936054)

5.1 Number of patients and target population [4](#__RefHeading___Toc444936055)

5.2 Inclusion criteria [4](#__RefHeading___Toc444936056)

5.3 Exclusion criteria [4](#__RefHeading___Toc444936057)

5.4 Discontinuation of treatment [5](#__RefHeading___Toc444936058)

5.5 Patient recruitment [5](#__RefHeading___Toc444936059)

5.6 Eligibility screening [5](#__RefHeading___Toc444936060)

5.7 Information to the family physician [6](#__RefHeading___Toc444936061)

6 Duration [6](#__RefHeading___Toc444936062)

6.1 Patients [6](#__RefHeading___Toc444936063)

6.2 Study [6](#__RefHeading___Toc444936064)

7 Ethics and good clinical practice [6](#__RefHeading___Toc444936065)

7.1 Declaration of Helsinki [6](#__RefHeading___Toc444936066)

7.2 Good Clinical Practice [6](#__RefHeading___Toc444936067)

7.3 Ethical procedures [7](#__RefHeading___Toc444936068)

7.4 Informed consent [7](#__RefHeading___Toc444936069)

7.5 Reporting and recording of data [7](#__RefHeading___Toc444936070)

8 Randomization [7](#__RefHeading___Toc444936071)

9 Medication [7](#__RefHeading___Toc444936072)

9.1 Medication characteristics [7](#__RefHeading___Toc444936073)

9.2 Dosing schedule [7](#__RefHeading___Toc444936074)

9.3 Production [8](#__RefHeading___Toc444936075)

9.4 Side effects [8](#__RefHeading___Toc444936076)

9.5 Concomitant medication [8](#__RefHeading___Toc444936077)

10 Results [8](#__RefHeading___Toc444936078)

10.1 Primary outcome measure [8](#__RefHeading___Toc444936079)

10.2 Secondary outcome measures [8](#__RefHeading___Toc444936080)

10.3 Measures taken in case of adverse events [9](#__RefHeading___Toc444936081)

11 Monitoring procedures [9](#__RefHeading___Toc444936082)

11.1 Monitoring of adverse events [9](#__RefHeading___Toc444936083)

11.2 Monitoring of compliance [9](#__RefHeading___Toc444936084)

12 Statistical considerations [9](#__RefHeading___Toc444936085)

12.1 Sample size [9](#__RefHeading___Toc444936086)

12.2 Stratification [10](#__RefHeading___Toc444936087)

12.3 Statistical methods [10](#__RefHeading___Toc444936088)

12.4 Interim analysis [10](#__RefHeading___Toc444936089)

13 Study records [10](#__RefHeading___Toc444936090)

13.1 Confidentiality [10](#__RefHeading___Toc444936091)

13.2 Storing of study records [10](#__RefHeading___Toc444936092)

14 Publication of results [10](#__RefHeading___Toc444936093)

15 Study organization and execution [11](#__RefHeading___Toc444936094)

15.1 Investigators [11](#__RefHeading___Toc444936095)

15.2 Study location [11](#__RefHeading___Toc444936096)

16 Reference List [12](#__RefHeading___Toc444936097)

# Study title

Effect of indomethacin on the progression of Alzheimer’s disease. A randomized double blind, placebo-controlled, multicenter clinical trial.

# Introduction

Inflammatory mechanisms are supposed to play an important role in the neuronal degeneration in patients with Alzheimer’s disease. This degeneration is associated with extracellular deposits of amyloid-β protein (Aβ) in senile plaques, abnormal phosphorylation of intraneuronal tau-proteins (neurofibrillary tangles) and clusters of activated microglial cells associated with senile plaques^1;2^.

Currently, international interest is directed to the role of activated microglial cells and their production of cytokines^2-4^. The acute phase protein α_1_-antichymotrypsin (α_1_-ACT), various complement factors (e.g. C1q), the intercellular adhesion molecule-1 (ICAM-1) and the cytokine interleukin-6 (IL-6) can be detected in senile plaques^5-8^. Increased activity of α_1_-ACT^9-12^ and decreased concentrations of IL-6 and C1q^13;14^ were found in cerebrospinal fluid of patients with Alzheimer’s disease. Presumably, activation of microglial cells occurs through interaction of Aβ proteins with microglial cells, mediated by a “receptor for advanced glycosylation end-products”(RAGE)^15^.

It is hypothesized that cytokines produced by activated microglial cells damage neurons and thus contribute to the deterioration of patients with Alzheimer’s disease. In this way, inhibition of production of cytokines would prevent the progression of the disease.

In retrospective epidemiological research the number of patients having arthritis and Alzheimer’s disease at the same time, was lower than to be expected from prevalence numbers of Alzheimer’s disease^16^. Presumably the use of nonsteroid anti-inflammatory drugs (NSAIDs) can account for this effect. Also, a twin study confirmed the decreased risk of Alzheimer’s disease when using NSAIDs^17^. A recent review of 17 epidemiological studies showed a consistent relation between the use of NSAIDs and a decreased incidence of Alzheimer’s disease^18^.

Based on these results a double blind, placebo-controlled trial was conducted with indomethacin in 44 patients with Alzheimer’s disease^19^. They concluded that patients in the indomethacin group deteriorated less rapidly than patients in the placebo group. However, because of the small number of patients studied, this trial should be reproduced.

The prospect of retarding the progression of Alzheimer’s disease with easily available and inexpensive drugs like NSAIDs is very attractive. Unfortunately, treatment with NSAIDs in the elderly is hampered by their toxicity and related serious side effects, including gastrointestinal bleeding and ulcers.

# Study objectives

## Primary objective

To assess whether indomethacin is able to retard disease progression in mild to moderate Alzheimer’s disease.

## Secondary objectives

- To evaluate the effect of indomethacin compared to placebo on cognitive and behavioral dysfunction and dysfunction in the activities of daily living in patients with mild to moderate Alzheimer’s disease.
- To evaluate the safety and tolerability of indomethacin.

# Study design

A randomized double blind, placebo-controlled, multicenter clinical trial to evaluate the effect of indomethacin over a one year period on the progression of mild to moderate Alzheimer’s disease. Following screening and baseline assessments, 160 patients will either receive indomethacin or placebo.

Evaluation of the ability of indomethacin to retard disease progression will be performed in weeks 26 and 52 (ADAS-cog/non-cog, MMSE, CIBIC+, NPI and IDDD). Safety en tolerability evaluations will be performed in weeks 4, 8, 26 and 52 (adverse events, physical examination, vital signs and laboratory evaluations). The evaluation schedule is specified in Appendix III.

# Patient selection

## Number of patients and target population

Approximately 160 patients with mild to moderate Alzheimer’s disease who satisfy inclusion and exclusion criteria will enter the study. Patients will be randomly assigned to either the medication or the placebo group (80 patients per group).

## Inclusion criteria

- The patient will satisfy the DSM-IV criteria for dementia of the Alzheimer’s type.
- The patient will satisfy the NINCDS/ADRDA criteria for the clinical diagnosis of probable Alzheimer’s disease (Appendix I)^20^.
- The severity of dementia for each patient will be quantified by a Mini-Mental State Examination (MMSE) score between 10 and 26 (both inclusive).
- The patient is living at home or in a home for the elderly.
- The patient has a responsible caregiver who is able to provide information about the patient’s functional status.
- Written informed consent is obtained from the patient or the legally accepted representative.

## Exclusion criteria

- The patient satisfies the NINDS-AIREN criteria for probable vascular dementia, including:
- presence of focal signs on neurological examination consistent with stroke
- evidence of relevant cerebrovascular disease by brain imaging (CT and/or MRI made within two years before the baseline visit)
- A known exaggerated pharmacological sensitivity or allergy to NSAID’s.
- History of peptic ulceration, gastric surgery or gastrointestinal bleeding.
- Current diagnosis of active peptic ulceration.
- Current diagnosis of severe and unstable cardiovascular disease.
- Current diagnosis of renal failure (creatinin > 200 μmol/l).
- Advanced, severe and unstable disease of any type, other than Alzheimer’s disease, that may interfere with primary and secondary variable evaluations, including a medical condition which should be expected to progress, recur, or change to such an extent that it may bias the assessment of the clinical or mental status of the patient to a significant degree or put the patient at special risk.
- Intake of any of the following concomitant medications:
- salicylates
- coumarin derivatives
- ACE-inhibitors
- loopdiuretics
- Intake of any of the following concomitant medications more than two months immediately prior or during the study:
- NSAID’s
- systemic corticosteroids
- Intake of any of the following concomitant medications with a possible effect on cognition:
- estrogen
- deprenyl
- vitamin E
- neuroleptics
- anticholinergics

Patients using stable doses of cholinesterase inhibitors were eligible, with the provision that the dose should not be changed during the study. Cholinesterase inhibitors could not be initiated during the study

- Excessive use of alcohol (> 5 units/day)
- The patient is, either alone or with the aid of a caregiver, not able to reliably take the medication.

## Discontinuation of treatment

- The patient decides to withdraw from the study.
- Presence of serious adverse events related to the study medication.
- Questionable compliance of the patient.
- Intake of systemic corticosteroids for more than two weeks.
- The patient develops a severe and unstable disease (see also exclusion criteria).
- The diagnosis of Alzheimer’s disease is no longer plausible considering the course of the disease.
- Breaking of the medication code.

## Patient recruitment

Patients will be recruited from the following populations:

- patients referred to the general outpatient clinic and the memory clinic of the Department of Geriatric Medicine, Radboud University Medical Centre Nijmegen
- patients referred to the outpatient clinic of the Department of Neurology, Radboud University Medical Centre Nijmegen
- patients referred to the memory clinic of the Department of Geriatric Medicine, Rijnstate Hospital, Arnhem

Recruitment through public media (e.g. local newspapers) will also be considered.

## Eligibility screening

Screening will be conducted at least 14 days before the patient begins taking study medication. Patients must satisfy the DSM-IV criteria for dementia of the Alzheimer type. Patients must also satisfy the NINCDS-ADRDA criteria for probable Alzheimer’s disease, have a MMSE score between 10 and 26 (both inclusive) and have a responsible caregiver to ensure compliance with the protocol.

To be eligible for the study, patients must satisfy selection criteria. Potentially eligible patients whose screening examinations show any disqualifying abnormality may have their examination to be repeated. Results must be within normal limits or judged not clinically significant before the patient starts treatment with the study medication.

Patients will undergo the following screening assessments (Appendix II): medical history, prior and concomitant medications or therapies, physical examination, vital signs, laboratory evaluations (including Apo E genotyping) and MMSE. Evaluation of the urine, electrocardiogram and CT scan of the brain will be conducted when not performed recently (CT scan > 2 years).

## Information to the family physician

When a patient is enrolled in the clinical trial, his family physician will be informed. The letter contains information about the purpose of the clinical trial, possible adverse events due to the study medication and telephone numbers to contact in case of emergency (Appendix I).

# Duration

## Patients

The study duration for the patient, from baseline to final evaluation, is one year. Six visits will take place in which different evaluations will be conducted. The evaluation schedule is specified in Appendix II.

## Study

The study duration, from recruitment of patients to the final analysis and reporting of results, will be four years. The following breakdown of this period should be considered:

- year 1: recruitment of 55 patients
- year 2: recruitment of 55 patients
- year 3: recruitment of 50 patients
- year 4: analysis and reporting of results

# Ethics and good clinical practice

## Declaration of Helsinki

The study will be conducted in accordance with the “Declaration of Helsinki”, concerning medical research in humans (“Recommendations Guiding Physicians in Biomedical Research Involving Human Subjects”, Helsinki 1964, amended Tokyo 1975, Venice 1983, and Hong Kong1989).

## Good Clinical Practice

The researchers will conduct the study following the standards of Good Clinical Practice (“Good Clinical Practice for Trials on Medicinal Products in the European Community”, CPMP Working Party on Safety of Medicinal Products, Brussels, 1990).

## Ethical procedures

The protocol and informed consent form will be presented to the Institutional Ethics Review Board (CMO) prior to the study initiation, who will have to provide a “statement of no objections”.

## Informed consent

All patients (if possible) and (if appropriate) a legally acceptable representative on the patient’s behalf and the patients caregiver will be fully informed about the study, orally and in writing. Each patient, if mentally competent, or a legally acceptable representative on the patient’s behalf, if appropriate, shall provide written consent before the patient may participate. Should the patient not be capable of providing written informed consent, written consent must be obtained from the legally acceptable representative on the patient’s behalf and verbal assent must be provided by the patient (Appendix III, IVa and IVb).

## Reporting and recording of data

Data on patients collected on clinical research forms (CRFs) in the course of this trial will be documented in a anonymous fashion (i.e. the patient will be identified only by a patient number and by patient initials). All information recorded on CRFs will be traceable back to the source documents, which are maintained in the patient’s file. The patient’s file will also indicate that he/she is participating in this clinical study.

# Randomization

Patients participating in the trial will be assigned a sequential trial number. The computer-generated list of trial numbers is linked to a randomized list with medication numbers, equivalent to 80 batches of indomethacin and 80 batches of placebo. The participating pharmacist will retain the randomization code. This procedure warrants a well-balanced initial randomization. However, a difference in the number of patients will occur when patients are withdrawn from the trial. The number of patients withdrawn from the indomethacin group will provide significant information in the final analysis of the data (“intention to treat”).

# Medication

## Medication characteristics

Indomethacin is a nonsteroidal anti-inflammatory drug (NSAID) used in the treatment of arthritis and other inflammatory disorders. It is no longer the most frequently used NSAID. However, it has the following characteristics favorable for use in Alzheimer’s disease:

- large volume of distribution
- good blood-brain barrier permeability
- results can be compared with the results from previously conducted studies

## Dosing schedule

Patients will receive indomethacin 50 mg bid. Omeprazole 20 mg will be prescribed in combination with the study medication (indomethacin or placebo), to provide gastrointestinal protection.

## Production

The amount of study medication required is 116.800 capsules of indomethacin 50 mg /placebo and 58.400 capsules of omeprazole. The generic medication will be ordered from a pharmaceutical company.

## Side effects

The risk for gastrointestinal side effects related to the use of NSAIDs in elderly people is 20 percent. In patients of 50 years and older, using a high dosage of the NSAID indomethacin and using corticosteroids at the same time, the risk of serious gastrointestinal complications can be 10 times higher, especially during the first three months. To prevent possible gastrointestinal side effects, omeprazol (20 mg once a day) will be prescribed^21^.

The patient’s discomfort will be small when no inconvenient side effects occur. A (small) discomfort for the patient will be the puncture in order to get blood samples.

## Concomitant medication

The patient’s concomitant medication will be maintained. Certain medication will lead to exclusion from the trial (see *5.3 exclusion criteria*).

# Results

## Primary outcome measure

Cognitive subscale of the Alzheimer’s Disease Assessment Scale (ADAS-cog):

The Alzheimer’s Disease Assessment Scale (ADAS) is a rating scale specifically designed for the evaluation of severity of major dysfunctions in cognitive (ADAS-cog) and noncognitive (ADAS-noncog) behaviors characteristic of persons with Alzheimer’s disease. It has become an assessment standard for use in clinical trials of new drugs being evaluated for the treatment of Alzheimer’s disease^22^.

The cognitive subscale of the ADAS evaluates the severity of cognitive dysfunction and consists of nine cognitive items (maximum score of 48 points) and two memory tasks (22 points), with a maximum total score of 70 points. The power calculation of this trial is based on this scale.

## Secondary outcome measures

Noncognitive subscale of the Alzheimer’s Disease Assessment Scale (ADAS-noncog): The noncognitive subscale evaluates the severity of noncognitive behavioral dysfunction. It consists of 10 items and a maximum score of 50 points.

Clinician Interview-Based Impression of Change with caregiver input (CIBIC+): The CIBIC+ is a global assessment instrument intended to provide an index of *clinically* important change that cannot be obtained from quantitative assessment measures such as mental status examinations (range 1 to 7)^23^.

Mini-Mental State Examination (MMSE): The MMSE is the most widely used screening instrument in Alzheimer’s disease drug studies. It is a short assessment, evaluating memory, attention, concentration, naming, repetition, comprehension, and the capacity to create a sentence. The MMSE is also frequently used to stratify patients according to disease severity. A perfect score on the MMSE is 30 and is influenced by educational achievement.

Neuropsychiatric Inventory (NPI): The NPI was developed to assess the severity and frequency of psychopathology (12 items) in dementia patients. The NPI-D assesses the amount of caregiver distress engendered by each of the neuropsychiatric disorders.^24^

The Interview for Deterioration in Daily living activities in Dementia (IDDD): The IDDD is a caregiver-based measure, which consists of 20 concretely worded items, reflecting the initiative to perform and actual performance of self-care and more complex activities.^25^

## Measures taken in case of adverse events

An adverse event is any undesirable sign, symptom or medical condition occurring after starting the intake of the trial medication, whether considered drug-related or not. Medical conditions/diseases present before starting the trial medication are only considered adverse events if they worsened after starting the medication.

In case of an adverse event with a possible causal relationship to the use of indomethacin, the medical attendant (e.g. family physician, physician) will discontinue the trial medication. The trial ends, however the trial code will not be broken and the patient’s data will be analyzed. The medical attendant will be asked to report this decision to the investigators as soon as possible.

The medical attendant is allowed to lower the dose of the study medication, in case of a dubious causal relationship between the adverse event and the study medication. The trial code will not be broken and the patient will be considered a normal participator of the trial.

In case of a serious adverse event, the patient’s medical attendant will ask the pharmacist to announce the nature of trial medication. The code will then be broken, the medication will be discontinued and the patient’s data will not be included in the analysis of the final outcome measures. However, the data of patients discontinuing the trial will analyzed according to the “intention to treat” principle.

# Monitoring procedures

## Monitoring of adverse events

Adverse events, whether volunteered by the patient, discovered during questioning by the investigator, or detected through physical examination (vital signs, symptoms of edema) or laboratory evaluation (haemoglobin, white blood cell count, platelet count, creatinine, glucose, liver parameters), will be recorded on the Adverse Event case report form and followed carefully until they resolve. Abnormal laboratory values or test results are not generally considered adverse events, unless they induce clinical signs or symptoms or require therapeutic intervention. An interim analysis of adverse vents will take place twice a year (see *12.4 Interim analysis*).

## Monitoring of compliance

The investigator will record the amount of dispensed trial medication. At each scheduled visit, all returned medication will be examined by the investigator to determine the number of unused capsules, and to determine if the medication used conforms to the amount expected. The information on missing doses will be recorded on the CRF. Also, the patient and caregiver will be questioned about the intake of trial medication.

# Statistical considerations

## Sample size

For estimation of the required sample size, the following assumptions were made:

- patients deteriorate 5.0 points (SD 7.0) on the ADAS-cog in one year.
- a difference of 3.0 points or more regarding the changes from baseline is considered to be clinically relevant in case indomethacin is tested against placebo.
- power of 80 percent.
- statistical testing will be done one-sided, at a significance level of 0.05.

Based on the above statistical considerations, a total of 67 patients per group will be necessary. Assuming a dropout rate of 20 percent, the total sample size will be 160 patients (80 patients per group).

## Stratification

During the initial randomization there is no use for further stratification. In the final analysis of the results, the indomethacin and placebo group will be compared concerning age, sex, duration and severity of the disease (ADAS-cog score) and Apo E phenotype.

## Statistical methods

In the final analysis of the results a so-called “evaluation of increment” will take place. Change in ADAS-cog score (primary outcome measure) of every patient will be compared between two groups by means of a 2 x 2 ANOVA (categories: group, time; α = 0.05).

Secondary outcome measures will be analyzed following the same procedure, with correction for multiple equations.

## Interim analysis

An interim analysis of the intended effect of indomethacin will not be conducted, considering the size of the studied population and the size of the expected effect.

# Study records

## Confidentiality

Study records will be considered medical information and thus confidentiality should be maintained. Should knowledge of patient identity become necessary for safety or regulatory reasons, confidentiality by the Investigator will be maintained.

## Storing of study records

During the study term the study records will be stored appropriately to ensure their confidentiality. They will remain in the possession of the investigator and must be kept for period of ten years.

# Publication of results

The study results will be presented to peer-reviewed journals for publication. All of the contributors named in the study proposal will be listed as co-authors. Anonymity and privacy of the patients will be guaranteed. If results are relevant to the society, the department “PR en Voorlichting” will be contacted for publication in the public media.

# Study organization and execution

## Investigators

Principal Investigators:

H.P.H. Kremer, M.D., Ph.D.

R.W.M.M. Jansen, M.D., Ph.D.

Coordination:

D. de Jong, M.D.

Development of the study protocol:

W.H.L. Hoefnagels, M.D., Ph.D.

R.W.M.M. Jansen, M.D., Ph.D.

H.P.H. Kremer, M.D., Ph.D.

D. de Jong, M.D.

M. Verbeek, Ph.D.

Y. Hekster, Ph.D.

M. van 't Hof, M.Sc., Ph.D.

Assessment of patients:

W.H.L. Hoefnagels, M.D., Ph.D.

R.W.M.M. Jansen, M.D., Ph.D.

D. de Jong, M.D.

M.J.H.Jellesma-Eggenkamp, M.D.

Patient recruitment:

D. de Jong, M.D.

Monitoring of adverse events:

Patient’s investigator

Y. Hekster, Ph.D.

Coding and dispensing of trial medication:

Y. Hekster, Ph.D. (D. Burger, Ph.D.)

Statistical processing:

M. van ’t Hof, M.Sc., Ph.D. (G.F. Borm, M.Sc., Ph.D.)

## Study location

Primary assessment of patients will take place at the outpatient clinic for memory disorders at the Department of Geriatric Medicine, University Hospital Nijmegen. Following assessments will take place either at the outpatient clinic or at the patient’s home, depending on his/her condition.

# Reference List

1. Selkoe DJ. Alzheimer's disease: a central role for amyloid [see comments]. J.Neuropathol.Exp.Neurol. 1994;53:438-447.

2. Eikelenboom P, Zhan SS, van Gool WA, Allsop D. Inflammatory mechanisms in Alzheimer's disease. Trends.Pharmacol.Sci. 1994;15:447-450.

3. Klegeris A, Walker DG, McGeer PL. Activation of macrophages by Alzheimer beta amyloid peptide. Biochem.Biophys.Res.Commun. 1994;199:984-991.

4. McGeer PL, Rogers J, McGeer EG. Neuroimmune mechanisms in Alzheimer disease pathogenesis [see comments]. Alzheimer.Dis.Assoc.Disord. 1994;8:149-158.

5. Verbeek MM, Otte Holler I, Westphal JR, Wesseling P, Ruiter DJ, de Waal RM. Accumulation of intercellular adhesion molecule-1 in senile plaques in brain tissue of patients with Alzheimer's disease. Am.J.Pathol. 1994;144:104-116.

6. Abraham CR, Selkoe DJ, Potter H. Immunochemical identification of the serine protease inhibitor alpha 1-antichymotrypsin in the brain amyloid deposits of Alzheimer's disease. Cell 1988;52:487-501.

7. Eikelenboom P, Stam FC. Immunoglobulins and complement factors in senile plaques. An immunoperoxidase study. Acta Neuropathol.Berl. 1982;57:239-242.

8. Strauss S, Bauer J, Ganter U, Jonas U, Berger M, Volk B. Detection of interleukin-6 and alpha 2-macroglobulin immunoreactivity in cortex and hippocampus of Alzheimer's disease patients. Lab.Invest. 1992;66:223-230.

9. Harigaya Y, Shoji M, Nakamura T, Matsubara E, Hosoda K, Hirai S. Alpha 1-antichymotrypsin level in cerebrospinal fluid is closely associated with late onset Alzheimer's disease. Intern.Med. 1995;34:481-484.

10. Licastro F, Parnetti L, Morini MC, et al. Acute phase reactant alpha 1-antichymotrypsin is increased in cerebrospinal fluid and serum of patients with probable Alzheimer disease. Alzheimer.Dis.Assoc.Disord. 1995;9:112-118.

11. Matsubara E, Hirai S, Amari M, et al. Alpha 1-antichymotrypsin as a possible biochemical marker for Alzheimer-type dementia. Ann.Neurol. 1990;28:561-567.

12. Brugge K, Katzman R, Hill LR, Hansen LA, Saitoh T. Serological alpha 1-antichymotrypsin in Down's syndrome and Alzheimer's disease. Ann.Neurol. 1992;32:193-197.

13. Yamada K, Kono K, Umegaki H, et al. Decreased interleukin-6 level in the cerebrospinal fluid of patients with Alzheimer-type dementia. Neurosci.Lett. 1995;186:219-221.

14. Smyth MD, Cribbs DH, Tenner AJ, et al. Decreased levels of C1q in cerebrospinal fluid of living Alzheimer patients correlate with disease state. Neurobiol.Aging 1994;15:609-614.

15. Yan SD, Chen X, Fu J, et al. RAGE and amyloid-beta peptide neurotoxicity in Alzheimer's disease [see comments]. Nature 1996;382:685-691.

16. Andersen K, Launer LJ, Ott A, Hoes AW, Breteler MM, Hofman A. Do nonsteroidal anti-inflammatory drugs decrease the risk for Alzheimer's disease? The Rotterdam Study. Neurology 1995;45:1441-1445.

17. Breitner JC, Gau BA, Welsh KA, et al. Inverse association of anti-inflammatory treatments and Alzheimer's disease: initial results of a co-twin control study. Neurology 1994;44:227-232.

18. McGeer PL, Schulzer M, McGeer EG. Arthritis and anti-inflammatory agents as possible protective factors for Alzheimer's disease: a review of 17 epidemiologic studies [see comments]. Neurology 1996;47:425-432.

19. Rogers J, Kirby LC, Hempelman SR, et al. Clinical trial of indomethacin in Alzheimer's disease. Neurology 1993;43:1609-1611.

20. McKhann G, Drachman D, Folstein M, Katzman R, Price D, Stadlan EM. Clinical diagnosis of Alzheimer's disease: report of the NINCDS-ADRDA Work Group under the auspices of Department of Health and Human Services Task Force on Alzheimer's Disease. Neurology 1984;34:939-944.

21. Gabriel SE, Jaakkimainen L, Bombardier C. Risk for serious gastrointestinal complications related to use of nonsteroidal anti-inflammatory drugs. A meta-analysis. Ann.Intern.Med. 1991;115:787-796.

22. Rosen WG, Mohs RC, Davis KL. A new rating scale for Alzheimer's disease. Am.J.Psychiatry 1984;141:1356-1364.

23. Knopman DS, Knapp MJ, Gracon SI, Davis CS. The Clinician Interview-Based Impression (CIBI): a clinician's global change rating scale in Alzheimer's disease. Neurology 1994;44:2315-2321.

24. Kat MG, de Jonghe JF, Aalten P, Kalisvaart CJ, Droes RM, Verhey FR. Neuropsychiatric symptoms of dementia: psychometric aspects of the Dutch Neuropsychiatric Inventory (NPI). Tijdschr.Gerontol.Geriatr. 2002;33:150-5.

25. Teunisse S, Derix MM. The interview for deterioration in daily living activities in dementia: agreement between primary and secondary caregivers. Int Psychogeriatr 1997;9 Suppl 1:155-62.

**APPENDIX I INFORMATION TO THE FAMILY PHYSICIAN**

Betreft: naam patiënt

adres

geboortedatum

Geachte collega,

Bovengenoemde patiënt neemt deel aan een dubbelblind, placebogecontroleerd onderzoek naar het effect van indometacine op het geheugen en algemeen dagelijks functioneren bij patiënten met de ziekte van Alzheimer. Gedurende twaalf maanden zal uw patiënt twee maal daags 50 mg indometacine dan wel placebo gebruiken. Daarnaast wordt een maal daags 20 mg omeprazol voorgeschreven om gastro-intestinale bijwerkingen te voorkomen. In verband met het onderzoek en eventuele bijwerkingen bij het gebruik van indometacine zal uw patiënt regelmatig op de polikliniek Geriatrie worden gecontroleerd.

Op grond van eerder verricht epidemiologisch onderzoek verwachten wij dat anti-inflammatoire middelen de progressie van de ziekte van Alzheimer remmen. Desgewenst zijn wij graag bereid meer informatie te verstrekken omtrent de achtergronden.

Mochten er zich onverwacht problemen voordoen, waarbij het noodzakelijk is te weten of uw patiënt indometacine dan wel placebo gebruikt, dan kunt u te allen tijde contact opnemen met ondergetekenden of de dienstdoende klinisch geriater via onderstaand telefoonnummer. De randomisatiecode kan dan eventueel worden verbroken.

Uw patiënt heeft randomisatienummer: …

Met collegiale hoogachting,

mede namens dr. H.P.H. Kremer, neuroloog,

Mw. D. de Jong, Dr. R.W.M.M. Jansen,

arts-onderzoeker klinisch geriater

Seinnummer *2265 Seinnummer *1050

Telefoonnummer (buiten kantooruren): 024-3616776

**APPENDIX II**

**Evaluation Schedule**

| **Evaluations** | **Screening** | **Baseline** | **Week 4** | **Week 8** | **Week 26** | **Week 38** | **Week 52** |
| --- | --- | --- | --- | --- | --- | --- | --- |
|  | **Week -2** | **Week 0** |  |  |  |  |  |
| NINDS/ADRDA criteria | X |  |  |  |  |  |  |
| DSM-IV criteria | X |  |  |  |  |  |  |
| MMSE | X | X |  |  | X |  | X |
| CT/MRI-brain | (X) |  |  |  |  |  |  |
| NINDS/AIREN criteria | X | (X) |  |  |  |  |  |
| Medical History | X |  |  |  |  |  |  |
| Medication | X |  |  |  |  |  |  |
| Physical Examination | X |  | X |  | X |  | X |
| Blood pressure/weight | X | X | X |  | X |  | X |
| Blood test | X |  | X | X | X |  | X |
| APO-E phenotyping | (X) |  |  |  |  |  |  |
| EKG | (X) |  |  |  |  |  |  |
| In/exclusion criteria | X | (X) |  |  |  |  |  |
| Informed consent |  | X |  |  |  |  |  |
| Randomisation number |  | X |  |  |  |  |  |
| ADAS-cog |  | X |  |  | X |  | X |
| ADAS-non cog |  | X |  |  | X |  | X |
| CIBIC+ |  | X |  |  | X |  | X |
| NPI |  | X |  |  | X |  | X |
| IDDD |  |  |  |  |  |  |  |
| Adverse events checklist |  |  | X | T | X | T | X |
| Distribution of indomethacin |  | X | X |  | X |  |  |
| Distribution of omeprazole |  | X | X |  | X |  |  |
| Indomethacin diary |  | X | X |  | X |  | X |
| Change in concomitant medication? |  | X | X | T | X | T | X |
| Change in medical condition? |  | X | X | T | X | T | X |

T = assessment by telephone

**APPENDIX III INFORMATION FOR THE PATIENT (Dutch)**

**Onderzoek naar het effect van het geneesmiddel indometacine op het beloop van de ziekte van Alzheimer.**

Inleiding

U bent gevraagd om deel te nemen aan een wetenschappelijk onderzoek naar het effect van een geneesmiddel met de naam indometacine. Indometacine is geen nieuw middel, want het wordt al heel lang door vele patiënten met reuma gebruikt. Uit diverse onderzoeken bij reumapatiënten is echter gebleken, dat dit geneesmiddel mogelijk het proces van de ziekte van Alzheimer vertraagt. Zodoende is bij een klein aantal Alzheimer patiënten het effect van dit geneesmiddel onderzocht, waarbij dit vermoeden werd bevestigd.

Doel van het onderzoek

Het doel van dit wetenschappelijk onderzoek is te bestuderen of het gebruik van indometacine de voortgang van de ziekte van Alzheimer kan remmen, bij patiënten met lichte tot matig ernstige verschijnselen van die aandoening. Om met behulp van deze onderzoeksopzet betrouwbare uitspraken te kunnen doen, zijn in totaal ongeveer 160 patiënten nodig, die gedurende één jaar het medicijn gebruiken.

Verloop van het onderzoek

Voorafgaand aan dit onderzoek wordt u uitvoerig onderzocht op de polikliniek Geriatrie. Er wordt lichamelijk onderzoek, bloed- en urinetesten gedaan, er volgt een CT-scan van de hersenen (indien deze nog niet eerder is verricht) en een psychologisch onderzoek van de geheugenfuncties. Als alle uitslagen goed zijn, maar er wel sprake is van de ziekte van Alzheimer, kunt u aan het onderzoek deelnemen.

Vervolgens zullen tijdens het gebruik van het geneesmiddel nog ongeveer vijf bezoeken (gedurende één jaar) aan de polikliniek volgen, om enkele testen te doen die te maken hebben met onder andere geheugen, oriëntatie en concentratie. Er wordt dan ook gevraagd naar lichamelijke klachten, er wordt lichamelijk onderzoek gedaan en het bloed wordt gecontroleerd op mogelijke bijwerkingen van het geneesmiddel.

Het geneesmiddel

Gedurende één jaar zult u capsules, met of zonder indometacine, gaan gebruiken. De helft van de patiënten krijgt dus capsules die geen werkzame stof bevatten (placebo capsules). Dit is nodig om na afloop van de studie betrouwbare uitspraken te kunnen doen over het werkelijke effect van het medicijn.

Er bestaat een kans dat u bijwerkingen krijgt van indometacine. De bijwerkingen die het meest optreden zijn misselijkheid, diarree, verminderde eetlust, hoofdpijn en duizeligheid. Omdat tevens bekend is dat het maagbloedingen kan geven, krijgt u er een medicijn bij dat de maag zoveel mogelijk beschermt en waarmee we deze bijwerking hopen te voorkomen. Mocht u toch symptomen van een maagbloeding hebben, bijvoorbeeld zwarte ontlasting, dient u onmiddellijk contact op te nemen met de onderzoekers of de dienstdoende klinisch geriater via onderstaand telefoonnummer.

Voorwaarden voor deelname

Om vast te stellen of u aan het onderzoek kunt deelnemen zijn er een aantal voorwaarden opgesteld die vooral te maken hebben met uw algehele conditie, eventuele bijkomende ziekten, gebruik van bepaalde medicijnen, uw woonsituatie en de aanwezigheid van een partner of vaste begeleider.

Ongemakken

De risico's die aan dit onderzoek verbonden zijn hangen samen met de mogelijke bijwerkingen van het medicijn en eventuele complicaties bij afnemen van bloed.

Vergoeding

De reiskosten die gemaakt worden voor deelname aan deze studie worden vergoed.

Uw rechten

Het onderzoek is beoordeeld door de Commissie voor Wetenschappelijk Onderzoek met Mensen van het St. Radboud Ziekenhuis te Nijmegen. Indien u deelneemt aan het onderzoek wordt u gevraagd een verklaring te ondertekenen waarin uw rechten en plichten zijn vastgelegd. Bij eventuele schade als gevolg van het onderzoek kunt u het ziekenhuis aansprakelijk stellen. Het ziekenhuis is hiervoor verzekerd.

Berichtgeving aan de huisarts

Vanaf het moment dat u deelneemt aan dit wetenschappelijke onderzoek wordt uw huisarts daarvan op de hoogte gesteld.

Vragen

Mocht u voor of tijdens deelname aan het onderzoek nog vragen hebben, dan kunt u contact opnemen met de onderzoekers (Mw. D. de Jong, arts-onderzoeker en dr. R.W.M.M. Jansen, klinisch geriater) of met de dienstdoende klinisch geriater (buiten kantooruren) via de polikliniek Geriatrie, telefoonnummer 024-3636776.

**APPENDIX IVa INFORMED CONSENT PATIENT (Dutch)**

**Onderzoek naar het effect van het geneesmiddel indometacine op het beloop van de ziekte van Alzheimer.**

Mijn behandelend arts, ……………….. heeft mij uitgelegd wat de voor- en nadelen, risico's en ongemakken van bovenvermeld onderzoek zijn. Ik heb de schriftelijke informatie gelezen en de gelegenheid gehad om vragen te stellen. Ik heb redelijk de tijd gehad om een en ander te overdenken. Ik begrijp wat de aard en het doel van dit onderzoek is.

Ik begrijp, dat deelname aan het onderzoek vrijwillig is en dat ik mij op elk moment zonder opgave van redenen uit dit onderzoek kan terugtrekken. Als ik dit doe, zal dit geen enkele invloed hebben op de voor mijn ziekte gebruikelijke behandeling en op de zorg van mijn behandelend arts.

Ik weet, dat voor dit onderzoek relevante medische gegevens over mij gebruikt worden voor wetenschappelijk onderzoek en eventueel gepubliceerd worden. Hiermee stem ik in, mits mijn privacy gewaarborgd wordt.

Mijn behandelend arts, ...............…...mag ter controle van de verzamelde gegevens inzage in relevante delen van mijn medische dossier verstrekken aan andere daartoe bevoegde autoriteiten, op voorwaarde dat hij/zij er voor in staat, dat de vertrouwelijkheid van deze gegevens niet zal en kan worden geschonden door deze personen.

Ik stem toe deel te nemen aan bovengenoemd onderzoek:

Achternaam en voorletters: ………………………………

Geboortedatum: ….. /….. /…..

Handtekening:

Datum: …../…../…..

Ondergetekende verklaart dat de hierboven genoemde patiënt over het bovenvermelde onderzoek geïnformeerd is:

Naam: ………………………………

Functie: ………………………………

Handtekening:

Datum: …../…../…..

**APPENDIX VIb** **INFORMED CONSENT LEGAL REPRESENTATIVE (Dutch)**

**Onderzoek naar het effect van het geneesmiddel indometacine op het beloop van de ziekte van Alzheimer.**

De behandelend arts, ……………….. heeft mij uitgelegd wat de voor- en nadelen, risico's en ongemakken van bovenvermeld onderzoek zijn. Ik heb de schriftelijke informatie gelezen en de gelegenheid gehad om vragen te stellen. Ik heb redelijk de tijd gehad om een en ander te overdenken. Ik begrijp wat de aard en het doel van dit onderzoek is.

Ik begrijp, dat deelname aan het onderzoek vrijwillig is en dat men zich op elk moment zonder opgave van redenen uit dit onderzoek kan terugtrekken. Als men dit doet, zal dit geen enkele invloed hebben op de voor de ziekte gebruikelijke behandeling en op de zorg van de behandelend arts.

Ik weet, dat voor dit onderzoek relevante medische gegevens gebruikt worden voor wetenschappelijk onderzoek en eventueel gepubliceerd worden. Hiermee stem ik in, mits de privacy gewaarborgd wordt.

De behandelend arts, ...............…...mag ter controle van de verzamelde gegevens inzage in relevante delen van het medische dossier verstrekken aan andere daartoe bevoegde autoriteiten, op voorwaarde dat hij/zij er voor in staat, dat de vertrouwelijkheid van deze gegevens niet zal en kan worden geschonden door deze personen.

Mij is gevraagd om toestemming te verlenen voor deelname van:

Achternaam en voorletters: ………………………………

Geboortedatum: …../…../….

aan bovenvermeld onderzoek.

Ik stem toe met deelname van bovengenoemde persoon aan dit onderzoek:

Achternaam en voorletters: ………………………………

Relatie tot de deelnemer: ………………………………

Handtekening:

Datum: …../…../…..

Ondergetekende verklaart dat de hierboven genoemde personen over het bovenvermelde onderzoek geïnformeerd zijn:

Naam: ………………………………

Functie: ………………………………

Handtekening:

Datum: …../…../…..
